# Deep-Coverage MPS Analysis of Heteroplasmic Variants within the mtGenome Allows for Frequent Differentiation of Maternal Relatives

**DOI:** 10.3390/genes9030124

**Published:** 2018-02-26

**Authors:** Mitchell M. Holland, Kateryna D. Makova, Jennifer A. McElhoe

**Affiliations:** 1 Department of Biochemistry & Molecular Biology, Forensic Science Program, Eberly College of Science, Pennsylvania State University, University Park, PA 16802, USA; jam760@psu.edu; 2 Department of Biology, Eberly College of Science, Pennsylvania State University, University Park, PA 16802, USA; kmakova@bx.psu.edu

**Keywords:** heteroplasmy, tissue differentiation, forensic science, next generation sequencing

## Abstract

Distinguishing between maternal relatives through mitochondrial (mt) DNA sequence analysis has been a longstanding desire of the forensic community. Using a deep-coverage, massively parallel sequencing (DCMPS) approach, we studied the pattern of mtDNA heteroplasmy across the mtgenomes of 39 mother-child pairs of European decent; haplogroups H, J, K, R, T, U, and X. Both shared and differentiating heteroplasmy were observed on a frequent basis in these closely related maternal relatives, with the minor variant often presented as 2–10% of the sequencing reads. A total of 17 pairs exhibited differentiating heteroplasmy (44%), with the majority of sites (76%, 16 of 21) occurring in the coding region, further illustrating the value of conducting sequence analysis on the entire mtgenome. A number of the sites of differentiating heteroplasmy resulted in non-synonymous changes in protein sequence (5 of 21), and to changes in transfer or ribosomal RNA sequences (5 of 21), highlighting the potentially deleterious nature of these heteroplasmic states. Shared heteroplasmy was observed in 12 of the 39 mother-child pairs (31%), with no duplicate sites of either differentiating or shared heteroplasmy observed; a single nucleotide position (16093) was duplicated between the data sets. Finally, rates of heteroplasmy in blood and buccal cells were compared, as it is known that rates can vary across tissue types, with similar observations in the current study. Our data support the view that differentiating heteroplasmy across the mtgenome can be used to frequently distinguish maternal relatives, and could be of interest to both the medical genetics and forensic communities.

## 1. Introduction

The advent of massively parallel sequencing (MPS) has paved the way for detailed analysis of mitochondrial (mt) DNA heteroplasmy in the fields of medicine [1,2], anthropology [3,4], and forensic science [5,6]. It has become increasingly clear that mutations in the human mtgenome are linked to a wide range of degenerative diseases, cancer, and aging [7,8,9,10,11], as the maternally inherited mtgenome codes for genes essential for the energy requirements of the cell, and for calcium buffering and sequestration. Given that mtDNA is present in hundreds to thousands of copies per cell, mutational events, deleterious or otherwise, typically pass through a heteroplasmic transition state [12]. Heteroplasmic variants migrate through a bottleneck in the female germline until fixation or elimination, and drift between and within somatic tissues through replicative segregation. The mechanism by which the variants become fixed in the germline is still poorly understood, but is essential for interpreting the clinical nature of resulting disease states. An empirically derived estimate of the size of the germline bottleneck in humans was calculated at ~30–35 copies of the mtgenome [13]. In another study, a variable-size model was used to estimate the mean bottleneck at nine copies [14], further illustrating that the size and consistency of the bottleneck remains unresolved [15]. Nonetheless, the restricted nature of the bottleneck clearly explains the dramatic drift in heteroplasmic variant ratios observed in several previous studies, including forensic investigations [6,16,17,18,19].

The identification of Nicholas Romanov, the last Russian Tsar, is an example of the power of forensic mtDNA analysis when including the interpretation of heteroplasmy [17]. When considering the haplotype match between the presumed skeletal remains of Nicholas and the known remains of his brother, Georgji, the findings were 150 times more likely, but increased to approximately 375,000 times more likely when including the shared heteroplasmy. The ratio of cytosine to thymine at position 16169 of the mtDNA control region (CR) [20] was approximately 2:1 for Nicholas, but 0.7:1 for Georgji. The narrow bottleneck exhibited in the development and maturation of individual oocytes from their mother, Maria Feodorovna, Princess Dagmar of Denmark and Empress of Russia, resulted in a major haplotype change from 16169C (Nicholas) to 16169T (Georgji). The estimated percentage of the C-variant in Nicholas was 67%, and 40% for Georgji; the former was verified through cloning experiments [21]. These findings illustrate the capacity of conventional Sanger-type sequencing (STS) to detect heteroplasmic variants that have reached at least 10-20% of the sequences in a DNA sample [22]. In addition, they emphasize that heteroplasmy is often shared by close maternal relatives when observed at higher levels. Contrary to this, apparent substitutions of one major haplotype for another have been observed between closely related individuals [23], illustrating that germline drift can be quite severe and further underscoring the weaknesses of STS for detecting low-level variants.

The transmission of heteroplasmic variants between maternal relatives is a relatively understudied area of mtDNA genetics when applying an MPS approach [13,24,25,26], including studies of twins [14] and centenarians [27]. A recent report on the analysis of mtgenomes from 194 mother-child pairs yielded no observations of differentiating heteroplasmy between the closely related individuals [28]. While 30 of the 194 pairs (15.5%) exhibited heteroplasmy, the sites of heteroplasmy were shared between the relatives, with the major haplotype shifting to the other variant in four instances. The MPS approach chosen by the authors [29] was the driving force for the findings, as read coverage on a per nucleotide basis was low (approximately 650 reads), with a requirement of 40 reads of the minor variant. As a result, at best, the threshold for detection of minor variants was approximately 6%. Despite this option, the authors set the reporting threshold at 10%. This approach significantly reduced the number of heteroplasmic sites captured by the MPS technique, as a relatively large percentage of the population has heteroplasmy between 2–10%; for example, more than 40% of the European population exhibits heteroplasmy within the CR alone, with approximately three quarters of the heteroplasmy between a frequency of 2–10% [30].

When assessing the transmission of heteroplasmy between maternal relatives using an MPS approach, two important considerations should be addressed. First, the depth of read coverage will dictate the sensitivity of detecting and resolving heteroplasmic sites. Ideally, a read depth of >2000 will allow for robust detection of variants at or below 2% [27,31]. Second, the tissue type chosen for studies of both rates of heteroplasmy and maternal inheritance of heteroplasmic variants will have an impact on the findings, so should be carefully considered when conducting these experiments. For instance, it is known that rates of heteroplasmy are greater in buccal cells than blood [13], and that kidney, liver, and skeletal muscle exhibit high rates of heteroplasmy [32]. Therefore, when conducting experiments on peripheral blood, and using an MPS approach with low read depths and high reporting thresholds for heteroplasmy [28], it is expected that little differentiating heteroplasmy will be observed, suggesting that this approach would not be useful in a forensic or clinical setting.

In the current study, an optimized method of deep-coverage, massively parallel sequencing (DCMPS) was used to evaluate the pattern of heteroplasmy in the mtgenomes of blood and buccal cells collected from 39 mother-child pairs. A threshold of 2% was used for reporting minor sequence variants, and error assessments were employed to ensure the reliability of the reported data. Two cell types were collected from the 39 pairs to assess tissues specific correlations, and to confirm the presence of differentiating heteroplasmy through analysis of cells originating from a different germ layer. Overall, our findings add to the growing body of knowledge regarding the pattern and rate of heteroplasmy within the mtgenome and could be of interest to both the medical genetics and forensic communities.

## 2. Materials and Methods

### 2.1. Samples and DNA Extraction

A total of 78 buccal and blood samples (156 samples total) were collected from mother-child pairs under an approved protocol from the Human Subjects Protection Office of the Pennsylvania State College of Medicine (IRB # 30432EP). The laboratory work conducted for this study was a collaborative effort between the Holland group (Forensic Science Program, Penn State University, University Park, PA, USA) and the Makova group (Biology Department, Penn State University, University Park, PA, USA). Collection of samples for the study, information about the study participants, and MPS data has been described previously [13,31]. The current study was a complete reanalysis of the data for the purposes of assessing the ability to differentiate maternal relatives. Extraction of DNA from buccal and blood cells was performed as described previously in Goto et al. [24].

### 2.2. Long-Range Polymerase Chain Reaction

The mtgenome was amplified using a long-range PCR approach with over-lapping 8.5 kilobase (kb) targets according to Goto et al. [24], and reported previously in [13,31]. In summary, the following oligonucleotide primer sets from Integrated DNA Technologies, Skokie, IL were used for amplification: 5’- GCGACCTCG-GAGCAGAAC-3’ (L2817) and 5’- GTAGGCAGATGGAGCTTG TTAT-3’ (H11570) for amplicon A, and 5’-CCACTGACATGACTTTCCAA-3’ (L10796) and 5’-AGAATTTTTCGTTCGGTAAG-3’ (H3370) [33] for amplicon B. One hundred nanograms (ng) of isolated genomic DNA (gDNA) was used as a template in a 50 μL PCR reaction containing 2 μM of each of the two primers, 200 μM dNTP (PCR grade; Roche Applied Science, Indianapolis, IN, USA), 3 units of Expand High Fidelity PCR Enzyme (Roche Applied Science), 1X PCR buffer with 1.5 mM MgCl, and nuclease-free water (Teknova, Hollister, CA, USA). The PCR parameters included a 94 °C soak for 2 min; followed by 10 cycles of 94 °C for 15 sec, 62.3 °C for 30 sec, and 68 °C for 8 min; followed by 30 cycles of 94 °C for 15 sec, 62.3 °C for 30 sec, and extension at 72 °C for 8 min. The extension time was elongated by 5 sec for each successive cycle during the last cycling phase. A final extension was performed at 72 °C for 7 min. Amplifications were carried out in a GeneAmp PCR System 9700 (ThermoFisher Scientific, USA). The PCR products (2 μL) were imaged by agarose gel electrophoresis to confirm successful amplification.

### 2.3. MiSeq MPS

All samples were sequenced on Illumina’s (San Diego, CA, USA) MiSeq benchtop sequencer, using Nextera^®^ XT (Illumina) sample preparation, and a 500-cycle reagent kit, and reported previously in [13,31]. In summary, samples were sequenced using paired 250 nucleotide reads, multiplexing 12, dual-indexed samples per run. Although the Nextera^®^ XT kit reagents were used in sample preparation, the protocol followed was a combination of the protocols available for Nextera^®^ XT and Nextera^®^ DNA kits. The manufacturers recommended protocol for Nextera^®^ XT was used with the exception of the bead normalization procedure. The bead normalization step streamlines library preparation for sequencing runs containing a large number of samples (i.e., multiplexing 96 samples), but each of the runs performed in this study contained 12 samples. Therefore, quantification and dilution of individual samples was a more efficient approach to normalizing the samples. Quantification and dilution is the method used in the Nextera^®^ DNA protocol for normalization, so this protocol was followed for the remainder of the library preparation. The sequences reported in this paper were deposited previously [13] in the Sequence Read Archive (SRA), www.ncbi.nlm.nih.gov/sra (accession no. SRP047378).

### 2.4. Data Analysis

All sequence data generated in this project was mapped to the revised Cambridge Reference Sequence (rCRS; GenBank ID NC_012920.1) [34] using MiSeq Reporter (Illumina v2.1.43 and v2.2.29), which uses a Burrows-Wheeler Aligner (BWA) and the Genome Analysis ToolKit (GATK) for variant calling of single nucleotide polymorphisms (SNPs) and short indels. Secondary analysis was performed on the existing dataset using GeneMarker^®^ High Throughput Sequencing (HTS) (GM-HTS; SoftGenetics, State College, PA, USA; v1.2.2) software [35]. The alignment algorithm in GM-HTS performs a Burrows-Wheeler [36] hash alignment based on spaced seeds (13 bases, ignore 1 base, and 13 more bases) and fills in gaps with dynamic programming. After alignment, a motif file (built-in or user-customized) can be applied to the reads. The motif file consists of a list of variant calls that are translated into an expected sequence. Each motif region is defined by a start and end nucleotide position and is inclusive, meaning that reads that do not span the entire region are trimmed. Alignment of reads spanning a defined motif region is adjusted to match the expected alignment pattern. For this exercise, FASTQ files were mapped to the rCRS using the following alignment options: customized motif file, 85% identity, and soft clipping at locations with three consecutive base pairs with a quality score ≤29. Table report settings were as follows: input region nucleotide position (np) 1–16,569, variant percentage ≥1% as the analytical threshold, variant allele coverage ≥40, total coverage ≥200, allele balance ratio ≤2.5, and allele score balance ≤10. A reporting threshold of 2% was used for calling heteroplasmic positions. The motif file, a simple text file containing phylogenetically correct sequence motifs that instructs the software which alignments are preferred by the user, contained 127 motifs that included those collected from the literature [37,38], as well as user-defined motifs based on new sequence patterns observed in the dataset. This process constituted a reanalysis of the dataset using a different software approach, with quality assessment of the data having been performed previously [13].

### 2.5. Error and Coverage Assessment

A conservative estimation of the substitution error rate for each base call (A, C, T, G) and base position (1–16,569) across the mtgenome, was used to establish the level of background noise in the sequencing data; i.e., from the combined effects of library preparation, sequencing, and secondary alignment. The analysis was performed by evaluating the consensus statistic files for all samples (*n* = 156 samples) with no mutation filtering, and assuming any calls constituting less than or equal to 50% of the total reads at a given position were made in error. Since substitution errors are the main source of errors on the MiSeq [39,40], insertions and deletions, most of which occur in simple repeat sequences, were omitted from this analysis. The error assessment was done using a combination of the MacOS terminal emulator with the Unix shell Bash, version 3.2.57(1) [41], (Bash 2007) and RStudio, version 1.1.383 [42] (Team R 2016).

Due to a characteristic uneven distribution of coverage associated with MPS sequencing of the mtgenome, a depth of coverage assessment was completed to determine the proportion of nucleotides that could not be characterized at the 2% minor allele frequency threshold due to filtering parameters applied in the GM HTS software. In this study, the filter settings required a minimum of 40 variant calls for any alternative allele to be reported by the software. This means that a filter setting of 40 calls required a minimum coverage of 2000 reads to report calls at 2%. The depth of coverage at each nucleotide position was taken from consensus statistic reports generated by GM HTS, prepared for upload using Terminal, and uploaded into RStudio for depth of coverage calculations. Note: contamination assessments were addressed previously [13].

### 2.6. Haplogroup Generation

Haplogroups were established using the collated major haplotype data and the Mitotool algorithm [43] incorporating Phylotree build 15 or 16 [44].

### 2.7. Statistical Analysis

Welch Two Sample *t*-tests were applied to evaluate the statistical significance of the heteroplasmy rate among shared, differentiating, and random sites of heteroplasmy, as well as a comparison of random heteroplasmy rates in mothers and children. All tests were conducted using RStudio.

## 3. Results and Discussion

### 3.1. Shared Heteroplasmy

A summary of the DCMPS results for the mother-child pairs can be found in Table 1, with additional details provided in Supplemental Tables S1–S3, including metadata associated with differentiating heteroplasmy (Table S2) and a comparison of shared, differentiating and random sites of heteroplasmy (Table S3). As a point of definition, shared heteroplasmy was considered when at least one of the two tissue types for both the mother and child of the pair shared the same site of heteroplasmy. Differentiating heteroplasmy was considered when one of the two family members had the same site of heteroplasmy in both buccal and blood samples, with the absence of the same site of heteroplasmy in the buccal and blood samples from the other family member. Lastly, random heteroplasmy was considered when sites were not replicated in the second tissue type of the same individual, and not observed in the other family member in a mother-child pair.

A total of 12 pairs (31%, 12 of 39) exhibited shared heteroplasmy, at 14 distinct sites across the 16,569 nps of the mtgenome. One family unit (No. 6, Table 1; No. 15, Table S1) had three sites of heteroplasmy, two of which resulted in a primary haplotype change between the mother and child. Two of the three sites were located in the coding portion of the mtgenome (nps 3243 and 5539), while the third site was located in the CR (np 16192) that resulted in length-based heteroplasmy at position 16191. The frequency of heteroplasmy at the three sites ranged from 13.13–41.94% in all four samples (buccal and blood from both mother and child), indicative of moderate genetic drift.

Two of the 12 mother-child pairs exhibited shared heteroplasmy in three of the four samples collected. In one family unit (No. 4, Table 1; No. 6, Table S1), the child had heteroplasmy of 6.66% and 5.40% in buccal and blood samples, respectively, while the mother had 2.53% heteroplasmy in her buccal cells, with no detectable heteroplasmy in her blood. This is an expected finding, as the genetic drift observed during development of different tissue types can routinely impact the ability to detect heteroplasmic variants when the overall frequency of heteroplasmy in the individual is low, even when using an MPS approach [6]. In the second family unit (No. 7, Table 1; No. 16, Table S1), the mother had heteroplasmy at np 16093 towards the SNP (i.e., T16093C heteroplasmy), with 11.53% heteroplasmy in buccal cells and 9.12% in her blood. The child exhibited 3.45% heteroplasmy in buccal cells, with no detectable heteroplasmy in the blood sample. In addition, the direction of the heteroplasmy for the child was towards the reference sequence (i.e., T16093T), illustrating that the mother and child differed in their primary haplotypes at np 16093. This is another example of both the severe germline bottleneck, as well as the potential drift between tissues types that can be observed when comparing sequence from maternal relatives possessing heteroplasmy.

The 14 distinct sites of shared heteroplasmy spanned the mtgenome, with 71% (10 of 14) occurring in the coding region. Of the 14 sites, four occurred in transfer RNA (tRNA) and ribosomal RNA (rRNA) genes, four sites resulted in synonymous changes to protein sequence, and two sites resulted in non-synonymous changes (Table S2). This is consistent with previous findings regarding the selection (cleansing) of potentially deleterious variants from the heteroplasmic pool [32]. Interestingly, in the current study a large region void of heteroplasmic sites was observed from nps 6153-10872, including the latter portion of the cytochrome oxidase 1 gene, all of cytochrome oxidase 2 and 3, ATP synthase 6 and 8, and NADH dehydrogenase 3, along with multiple tRNA genes. While potentially impactful, this finding is most likely due to sample size, as previous studies have found multiple sites of heteroplasmy throughout this region [28], albeit in a different population group (Han Chinese).

The findings above are consistent with a recent report on the analysis of mtgenomes from 194 mother-child pairs [28], where 30 of the 194 pairs (16%) exhibited shared heteroplasmy, and where the major haplotype shifted to the other variant in four instances. The difference in rates of shared heteroplasmy (16% v. 31%) can be attributed to the different approaches employed. The read coverage for each nucleotide was low (approximately 650 reads) for the previous study, with a requirement of 40 reads of the minor variant and a reporting threshold for heteroplasmy set at 10%. The current study had an average coverage rate of 24,335, with the same requirement of 40 reads for the minor variant, and a reporting threshold of 2%. Of the 12 pairs in the current study with shared heteroplasmy, three exhibited heteroplasmy below 10% for all samples tested, and another six pairs had at least one sample with heteroplasmy below 10%. If the low-coverage, high-threshold approach were applied to this data the rates of shared heteroplasmy would have dropped to 8–23%, which encompasses the rate from the previous study. As expected when considering shared heteroplasmy, 75% of the pairs in the current study (9 of 12 pairs) had heteroplasmy greater than 10% in one or more samples, with 25% of the pairs exhibiting heteroplasmy greater than 10% in all four tissue samples. In general, when levels of heteroplasmy are relatively high, it is expected that the heteroplasmy will be more often shared by maternal relatives. Most importantly, the ability to report heteroplasmic variants at a lower threshold when using a DCMPS approach clearly increases the likelihood of identifying sites of shared heteroplasmy, with this observation holding true for differentiating heteroplasmy.

### 3.2. Differentiating Heteroplasmy

A summary of the metadata associated with differentiating heteroplasmy for the 39 mother-child pairs can be found in Table S2, with comparisons to shared and random heteroplasmy in Table S3. A total of 17 pairs (44%) exhibited differentiating heteroplasmy (summarized in Table 2), defined as one of the two family members having the same site of heteroplasmy in both buccal and blood samples, with the absence of the same site of heteroplasmy in the buccal and blood samples from the other family member. Potential SNP-based heteroplasmy at np 310 was not included in the analysis. A total of 21 distinct sites of differentiating heteroplasmy were identified in the 17 pairs, with a single site of heteroplasmy observed in 14 of the pairs. In two family units (Nos. 6 and 15, Table 2; Nos. 10 and 23, Table S2), both the mother and child had a site of differentiating heteroplasmy. The location of these sites varied from the CR to both synonymous and non-synonymous changes to protein sequence, with three of the four sites located in the coding region. In a third family unit (No. 11, Table 2; No. 19, Table S2), the mother had a single site of differentiating heteroplasmy, while the child had two sites. All three sites were in the coding region; one non-synonymous change to the ATP synthase 6 gene, one change to the 16S rRNA sequence, and one change to the tRNA^thr^ gene. Based on these findings, it is quite possible that multiple sites of differentiating heteroplasmy will be observed when comparing the mtgenome sequences from maternal relatives; 18% of the families (3 of 17) exhibited multiple sites of differentiating heteroplasmy.

No significant correlation to the origin of the differentiating heteroplasmy was observed in our data, as nine of the 21 sites (43%) were limited to the child, while the remaining 12 sites were restricted to the mother. In addition, the rates of differentiating heteroplasmy were not well correlated to tissue type, consistent with how it was delineated in the current study; i.e., that the heteroplasmy had to be observed in both buccal and blood cells. Consistent with expectations, 91% of the sites (19 of 21) associated with buccal samples had higher rates of heteroplasmy when compared to blood samples. However, an assessment of the rates for each site revealed that most sites had a narrow range of variant frequencies between the two tissue types. The vast majority of sites (81%, 17 of 21) exhibited less than a twofold difference in the rate values. Exceptions included a family unit (No. 2, Table 2; No. 4, Table S2) with 5.07% heteroplasmy at np 16320 of the CR in the blood of the mother, with 27.57% heteroplasmy in her buccal sample; an approximately fivefold difference in rates. The remaining three exceptions ranged from a two to threefold change in rates.

Compared to shared heteroplasmy, the rate of differentiating heteroplasmy was more often observed in the range of 2–10% (Table S3). Approximately 67% of sites (14 of 21) exhibited differentiating heteroplasmy below 10% for each of the tissue types, while the frequency of heteroplasmy at only 5 of 21 sites (24%) was greater than 10% for both tissues. In contrast, only 21% of sites (3 of 14) with shared heteroplasmy had frequencies below 10%, while 36% (5 of 14) had greater than 10% heteroplasmy for all tissues tested. The average frequency of shared heteroplasmy across all samples was 15.6%, while the average for differentiating heteroplasmy was 8.6% (significantly lower, *p* = 9.2 × 10^−4^), emphasizing the importance of read depth and reporting thresholds when attempting to identify sites of differentiating heteroplasmy. Coverage for each position of both shared and differentiating heteroplasmy was greater than 2000, and typically greater than 10,000 reads allowing for the application of a DCMPS approach.

### 3.3. Random Heteroplasmy

A summary of the metadata associated with random heteroplasmy for 29 of the 39 mother-child pairs can be found in Table S2, with comparisons to shared and differentiating heteroplasmy in Table S3. Random heteroplasmy was defined as sites not replicated in the second tissue type of the same individual, and not observed in the other family member in a mother-child pair. These sites are presumed to originate from de novo mutations or low-level sites of heteroplasmy not detected in other tissues or the second individual in a pair. Potential SNP-based heteroplasmy at np 310 was not included in the analysis. A total of 20 of the 39 pairs (52%) exhibited random sites of heteroplasmy across the mtgenome, with an average of 0.77 heteroplasmies per pair, and 0.39 per individual. Of the 30 sites of random heteroplasmy, 24 sites were observed once, and three sites were observed twice; nps 215, 16093 and 16189. Fourteen of the 30 sites were located in the CR, while the remaining 16 (53%) were located in the coding region, once again highlighting the value of sequencing the entire mtgenome.

The frequency of the minor variant for random sites of heteroplasmy was significantly lower than the sites of shared (*p* = 2.2 × 10^−5^) and differentiating heteroplasmy (*p* = 5.0 × 10^−4^) and differentiating heteroplasmy was significantly lower than shared (*p* = 9.2 × 10^−4^). The average frequency was 3.8%, compared to 8.6% for differentiating heteroplasmy and 15.6% for shared heteroplasmy. A single site of random heteroplasmy (np 16093) had a frequency greater than 10%, while 13 of the 30 sites were between 3–10%, and 16 sites had a frequency between 2–3%. This is an expected outcome, as random heteroplasmy typically has not accumulated to a high level. Interestingly, of the 30 sites of random heteroplasmy, seven sites (23%) were observed in mothers, while 77% were found in children. These values are trending towards the younger of the two individuals in the pair but are not significantly different (*p* = 0.33) and reflect the inconsistent reporting of whether heteroplasmy is strongly correlated to age. In addition, an important consideration is the tissue type being tested. In the current study, 24 of the 30 sites of random heteroplasmy were observed in buccal samples, consistent with previous reports of elevated levels of heteroplasmy in buccal cells.

### 3.4. Coverage and Error Rates

Using the DCMPS approach for the current study, average coverage across the mtgenome was 24,335 reads per nucleotide position. The depth of coverage was >2000 reads for 98.7% of the nucleotide positions, allowing for the application of a 2% threshold given the requirement of 40 reads for a minor variant to be reported. Approximately one third of the samples tested (52/156) had a depth of coverage exceeding 2000 for all 16,569 nucleotide positions, another third had <100 nucleotide positions with coverage <2000, and the final third varied in the number of positions under 2000 reads. The majority of observations of coverage <2000 occurred between nps 301–530 and 3567–3572, a relatively narrow range of the mtgenome, although approximately 35% of the positions were affected at some level. Regions that produced low coverage generally did so due to the challenges of producing quality sequence data through certain regions of the mtgenome and the nature of the alignment strategy of the GM HTS software. For example, motif driven alignment resulted in a reduction in coverage for regions of homopolymeric and repetitive sequence, as trimming reduced the read depth in the range of sequence being aligned. Nonetheless, all mother-child pairs with differentiating heteroplasmy were manually evaluated to ensure that sites observed to have no heteroplasmy at corresponding nucleotide positions were not removed due to coverage-related filtering. In each case, sufficient read coverage to apply a 2% threshold was confirmed, and across each tissue type (Table 2).

To confirm that heteroplasmy was due to true signal above noise associated with amplification, library preparation, and sequencing on the MiSeq, the substitution-based error rate at each nucleotide position across the mtgenome was calculated for all nucleotides (A, C, G, and T). Greater than 60 billion total base calls were used for the analysis, taken from the quality-filtered dataset, as quality trimming has been shown to improve error rates [45]. Error profiles for MPS data are not well understood [40], and the available methods for error rate estimation are limited [46]. Therefore, our approach to calculating assumed substitution-based error was conservative, including all base calls with frequencies less than or equal to 50% in the calculation. Given that MiSeq calls have been shown to be concordant with traditional Sanger sequencing [31,47], the 50% cut-off represents a worse-case scenario and is marginally inflated due to the inclusion of known heteroplasmic variants.

**Figure 1:**
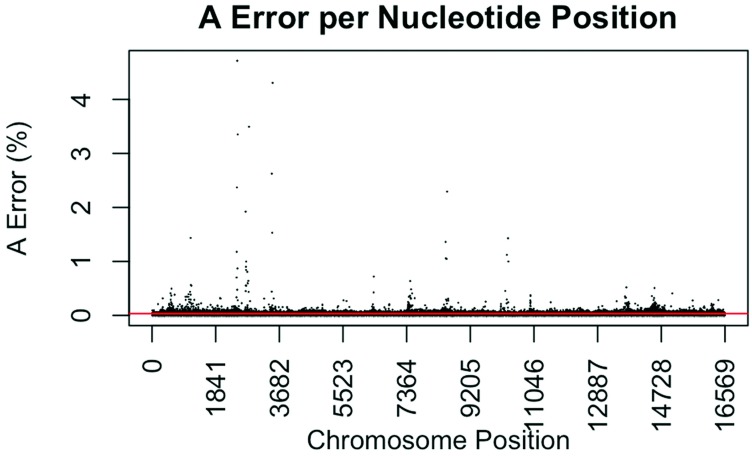
Substitution error rate for A (adenosine) nucleotides per nucleotide position across the mtgenome. The error rate was calculated by dividing the sum of all A calls assumed in error in all samples (all A calls observed at <50%) by the total number of calls (or reads) at that nucleotide position for all samples. The red line indicates the average A error (0.0343%) for all nucleotide positions.

**Figure 2:**
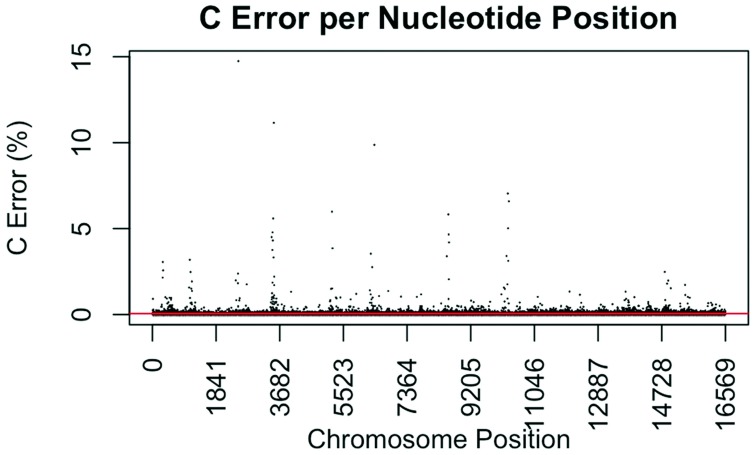
Substitution error rate for C (cytidine) nucleotides per nucleotide position across the mtgenome. The error rate was calculated as described in Figure 1. The red line indicates the average C error (0.0565%) for all nucleotide positions.

**Figure 3:**
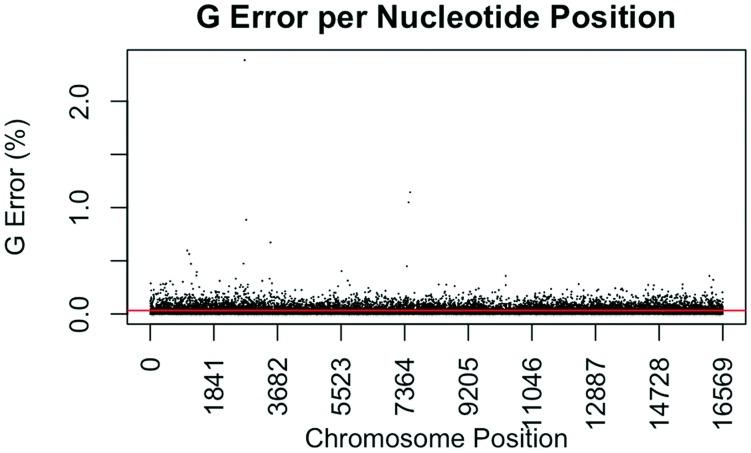
Substitution error rate for G (guanosine) nucleotides per nucleotide position across the mtDNA genome. The error rate was calculated as described in Figure 1. The red line indicates the average G error (0.0331%) for all nucleotide positions.

**Figure 4:**
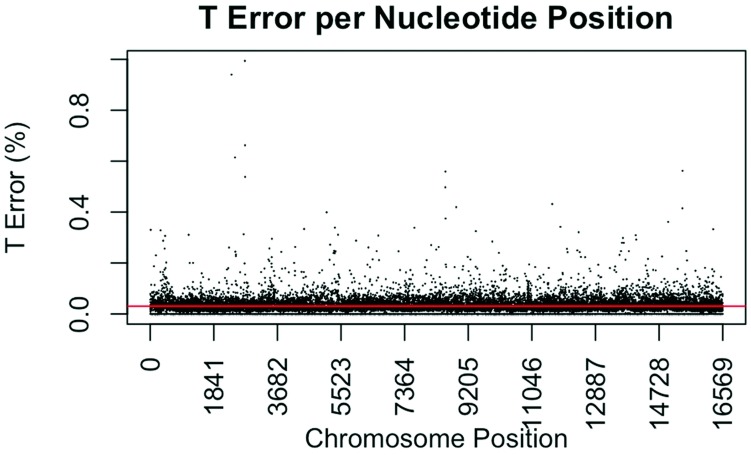
Substitution error rate for T (thymidine) nucleotides per nucleotide position across the mtgenome. The error rate was calculated as described in Figure 1. The red line indicates the average T error (0.0304%) for all nucleotide positions.

The consensus statistic reports generated by GM HTS were used to calculate error rates based on the number of times each nucleotide was called in relation to total coverage, and then filtered to include only calls with <50% frequency. Assumed error was then evaluated in two ways, looking at an average error rate and a rate based on each nucleotide position. For the average error, the number of calls for each nucleotide (A, C, G, and T) was summed across all samples for all 16,569 nucleotide positions and divided by the sum of coverage across all samples for all 16,569 positions. The observed error for each nucleotide position was calculated by summing the number of calls for each nucleotide across all samples at each position (1–16,569) and dividing the sum of the coverage across all samples at each nucleotide position. This generated 16,569 rates for A, C, G, and T (Figure 1, Figure 2, Figure 3 and Figure 4). The average assumed substitution-based error rate for each nucleotide was well below our threshold of 2% (Table 3), indicating that heteroplasmic positions reported at 2% are well above the noise of the system. The average rates indicate the system as a whole has low error, which has been shown by others [40,48,49], but this assessment does not provide information as to whether hotspots of error exist at specific locations across the mtgenome.

Our evaluation of error for each nucleotide indicates that the assumed rate of error varies across the mtgenome and supports previous studies [50]. The highest rates of error for each nucleotide (25 rates for A, G, and T, and 35 for C; to include all C rates >2%; 110 rates total) encompassed 105 positions with 99 of those positions falling within the coding region and nine positions within the CR. The estimated T error did not surpass 1.05% at any position in the mtgenome, while G error surpassed 2% at one position (np 2734), A error surpassed 2% at seven positions, and C error had the greatest number of sites, 31 nucleotide positions, surpassing 2% (Table 4). Even though 39 nucleotide positions had error greater than our 2% threshold, no observations of heteroplasmy were reported at these positions. Manual inspection of multiple sequencing pile-ups indicate that these locations, while having a mixture of nucleotides, are not reported as valid heteroplasmic positions due to failing both the balance ratio as well as the quality score filtering parameters. The underlying reason for these spikes in error was determined to be motif driven, occurring in only one direction, and therefore not reported due to read imbalance filtering. These positions also had lower quality scores for the minor variant, causing the position to fail the quality score filter. Other studies have associated error with similar emission spectra of A/C or G/T fluorophores [39,40,51] and specific sequence patterns such as GGT [52,53] or AAA [48]. Our error assessment supports previous findings with over half of the top error sites being located adjacent to specific sequence motifs: 19% (21/110) adjacent to a sequence motif of at least three A nucleotides, 30% (33/110) adjacent to at least three C nucleotides, and 6.3% (7/110) associated with a motif of GGT. Our observations also support previous findings [54] that the most frequent type of error was A>C or C>A transversions (32%). Other error sites consisted of A>T or T>A (26%), and G>T or T>G (16%) type errors. The error evaluation supports our reporting threshold of 2% when assessing minor sequence variants that represent heteroplasmies due to true signal above noise.

## 4. Conclusions

Based on the findings of the current study, the ability to differentiate maternal relatives when conducting forensic mtDNA analysis is clearly possible on a routine basis when using a DCMPS approach. While it is certainly ideal to target the entire mtgenome, as the majority of the differentiating heteroplasmy was observed in the coding region (76%), analysis of the CR may allow for the differentiation of maternal relatives in approximately one in seven forensic cases. Therefore, practitioners currently targeting the CR will still benefit greatly when choosing to implement the DCMPS approach, while deferring expansion of their analysis to the entire mtgenome.

The use of a DCMPS approach cannot be emphasized enough. Without sufficient depth of coverage at a nucleotide position, it is challenging to report heteroplasmy below the 10–20% threshold typically reserved for STS. The majority (71%) of the differentiating heteroplasmy in the current study was observed in the range of 2–10%, highlighting the need for a low reporting threshold. Tissue specific considerations are also important when assessing the potential usefulness of differentiating heteroplasmy. In the current study, differentiating heteroplasmy was not considered unless observed in both buccal and blood cells. In a forensic case, hair shafts found at a crime scene are a common source of biological material for analysis, while the reference source from a suspect or victim is typically a buccal sample. Previous studies have illustrated that for direct comparison purposes, buccal samples are a better choice for the reference sample when working with hairs, as they originate from the same germ layer [6]. However, it may be important to collect both a buccal and blood sample from the reference source when differentiation of maternal relatives is being considered in a case. This would allow for an assessment of whether the heteroplasmy is random or potentially differentiating.

This is an exciting time in the development of MPS-based methods for the analysis of mtDNA in forensic cases. The use of a DCMPS approach will have a positive impact on the ability to solve more cases, and to enhance the weight of the findings. In addition, the analysis of low-level heteroplasmic variants will enhance the ability of clinicians to diagnose health-related conditions and provide counseling services to expecting parents. Along the way, further work will be needed to assess the drift of low-level heteroplasmic variants between tissue types and across generations, and to evaluate the impact of different MPS platforms on interpretation of the data. In addition, it would be valuable to develop enhanced methods of software alignment that may provide for a continuum of error assessments across nucleotide positions, perhaps allowing for the lowering of reporting thresholds for certain positions.

## Figures and Tables

**Table 1:** Shared heteroplasmy for 12 of the 39 mother-child pairs. Frequency of heteroplasmy at each nucleotide position (np) in percentage (%), with the minor variant annotated as the letter after the np; i.e., A1656A (2.11%) is heteroplasmy at np 1656, with the first A as the reference sequence and the second A as the minor variant at 2.11% of the reads. Underlined letters following the np of heteroplasmy indicate a primary haplotype change between the mother and child. In two instances, heteroplasmy was not detected (ND) in a sample.

Mother-Child Pair	Sample Number	Shared Heteroplasmy
1	Mother-Bu (807)	A16183G (7.32%)
	Child-Bu (803)	A16183G (6.89%)
	Mother-Bl (M490)	A16183G (2.81%)
	Child-Bl (M490-C)	A16183G (2.46%)
2	Mother-Bu (618)	T16189C (7.74%)
	Child-Bu (606)	T16189C (11.07%)
	Mother-Bl (M249)	T16189C (2.81%)
	Child-Bl (M249-C)	T16189C (9.92%)
3	Mother-Bu (704)	T6152C (7.23%)
	Child-Bu (630)	T6152C (16.37%)
	Mother-Bl (M234)	T6152C (5.04%)
	Child-Bl (M234-C)	T6152C (16.48%)
4	Mother-Bu (762)	T10873C (2.53%)
	Child-Bu (702)	T10873C (6.66%)
	Mother-Bl (M210)	ND
	Child-Bl (M210-C)	T10873C (5.40%)
5	Mother-Bu (729)	A1656A (2.11%)
	Child-Bu (684)	A1656A (2.52%)
	Mother-Bl (M213)	A1656A (2.77%)
	Child-Bl (M213-C)	A1656A (2.68%)
6	Mother-Bu (1091)	A3243G (30.72%), A5539A (41.94%) and C16192C (19.23%)
	Child-Bu (1111)	A3243A (33.10%), A5539G (24.54%) and C16192C (14.10%)
	Mother-Bl (M512)	A3243G (13.13%), A5539A (23.13%) and C16192C (22.78%)
	Child-Bl (M512-C)	A3243A (41.01%), A5539G (31.26%) and C16192C (17.30%)
7	Mother-Bu (1098)	T16093C (11.53%)
	Child-Bu (1100)	T16093T (3.45%)
	Mother-Bl (M520)	T16093C (9.12%)
	Child-Bl (M520-C)	ND
8	Mother-Bu (1267)	T2352T (48.11%)
	Child-Bu (1160)	T2352T (26.81%)
	Mother-Bl (SC16)	T2352T (47.93%)
	Child-Bl (SC16-C)	T2352T (26.84%)
9	Mother-Bu (839)	C11635T (8.34%)
	Child-Bu (1189)	C11635T (17.93%)
	Mother-Bl (M494)	C11635T (7.23%)
	Child-Bl (M494-C)	C11635T (19.88%)
10	Mother-Bu (632)	G15047A (21.08%)
	Child-Bu (696)	G15047A (26.67%)
	Mother-Bl (M236)	G15047A (19.47%)
	Child-Bl (M236-C)	G15047A (28.22%)
11	Mother-Bu (531)	C5107T (9.74%)
	Child-Bu (572)	C5107T (13.07%)
	Mother-Bl (M-188)	C5107T (8.19%)
	Child-Bl (M188-C)	C5107T (10.05%)
12	Mother-Bu (616)	T15262C (8.36%)
	Child-Bu (643)	T15262C (15.81%)
	Mother-Bl (M252)	T15262C (7.46%)
	Child-Bl (M252-C)	T15262C (15.49%)

Bu: buccal cell; Bl: blood.

**Table 2:** Differentiating heteroplasmy for 17 of the 39 mother-child pairs; buccal cell (Bu) and blood (Bl) samples. Frequency of heteroplasmy at each np in percentage (%), with the minor variant annotated as the letter after the np; i.e., T2746C is heteroplasmy at np 2746, with 20.11% of the C variant. Coverage and read distribution (forward reads, #For, compared to reverse reads, #Rev) are provided for each np of differentiating heteroplasmy, along with the gene annotation and whether sites in protein coding genes result in a synonymous change (Y) or not (N). The gene annotations include: CR = control region, 12S & 16S = 12S & 16S rRNAs, ATP6 = ATP synthase 6, ND = NADH dehydrogenase, tRNA^thr^ = tRNA for threonine, and CO = cytochrome oxidase. Metadata for samples without the heteroplasmy are provided to illustrate that read percentages are clearly below reporting threshold and that coverage was adequate for this assessment.

Mother-Child Pair	Nucleotide Position	Sample Number	Major Allele	Coverage (#For:#Rev Reads)	Major Frequency (%)	Minor Allele	Coverage (#For:#Rev Reads)	Minor Frequency (%)	Gene Annotation	Synonymous (Y or N)
1	T2746C	Mother - Bu (693)	T	2920:6014	79.67	C	655:1600	20.11	16S	
		Child - Bu (677)	T	4838:14038	99.64	C	1:9	0.053		
		Mother - Bl (M207)	T	14187:14328	80.3	C	3440:3528	19.62	16S	
		Child - Bl (M207-C)	T	24044:24176	99.88	C	6:12	0.037		
2	C16320T	Mother - Bu (406)	C	4918:3843	72.33	T	1866:1474	27.57	CR	
		Child - Bu (444)	C	17616:13273	99.92	T	9:7	0.052		
		Mother - Bl (M137)	C	5412:4619	94.9	T	288:248	5.07	CR	
		Child - Bl (M137-C)	C	4232:3670	99.92	T	2:1	0.038		
3	T9179C	Mother - Bu (1134)	T	3063:5076	85.02	C	538:892	14.93	ATP6	N (Val to Ala)
		Child - Bu (1099)	T	6651:8730	99.82	C	8:7	0.097		
		Mother - Bl (M502G)	T	16583:20269	87.14	C	2468:2934	12.77	ATP6	N (Val to Ala)
		Child - Bl (M501)	T	38769:44060	99.81	C	32:24	0.067		
4	G14040A	Mother - Bu (659)	G	5770:4227	92.01	A	474:381	7.86	ND5	Y (Gln)
		Child - Bu (722)	G	20789:16141	99.86	A	8:12	0.054		
		Mother - Bl (M242)	G	13200:12992	94.07	A	831:811	5.89	ND5	Y (Gln)
		Child - Bl (M242-C)	G	10355:10087	99.88	A	5:5	0.049		
5	T14461C	Mother - Bu (411)	T	7078:7720	97.04	C	205:233	2.87	ND6	Y (Thr)
		Child - Bu (401)	T	16084:15992	99.78	C	35:18	0.165		
		Mother - Bl (M132)	T	8475:8875	97.54	C	193:237	2.41	ND6	Y (Thr)
		Child - Bl (M132-C)	T	5854:6622	99.92	C	8:1	0.072		
6	G11825A	Mother - Bu (711)	G	1622:2662	93.41	A	116:184	6.54	ND4	N (Ala to Thr)
		Child - Bu (737)	G	8943:15813	99.8	A	8:3	0.044		
		Mother - Bl (M203)	G	4728:5871	97.1	A	133:167	2.74	ND4	N (Ala to Thr)
		Child - Bl (M203-C)	G	14625:18730	99.88	A	6:5	0.033		
	T12375C	Mother - Bu (711)	T	1713:1597	99.67	C	1:9	0.301		
		Child - Bu (737)	T	6368:6238	72.03	C	2770:2099	27.82	ND5	Y (Thr)
		Mother - Bl (M203)	T	4588:4190	99.66	C	13:12	0.284		
		Child - Bl (M203-C)	T	10455:10132	76	C	3481:3008	23.95	ND5	Y (Thr)
7	A13790G	Mother - Bu (729)	A	2539:943	99.63	G	1:1	0.057		
		Child - Bu (684)	A	5501:2427	88.46	G	650:356	11.22	ND5	N (Tyr to Cys)
		Mother - Bl (M213)	A	10487:7516	99.46	G	4:25	0.160		
		Child - Bl (M213-C)	A	5900:4359	88.44	G	705:583	11.1	ND5	N (Tyr to Cys)
8	A200A	Mother - Bu (1098)	G	1350:3677	96.58	A	31:139	3.26	CR	
		Child - Bu (1100)	G	1148:3998	98.79	A	5:54	1.13		
		Mother - Bl (M520)	G	821:1294	97.6	A	17:32	2.26	CR	
		Child - Bl (M520-C)	G	5713:9174	99.59	A	14:41	0.368		
9	A4191T	Mother - Bu (1122)	A	8781:10930	99.36	T	2:27	0.146		
		Child - Bu (1119)	A	4452:5729	95.5	T	202:243	4.17	ND1	Y (Pro)
		Mother - Bl (M500)	A	5699:6469	99.37	T	3:25	0.229		
		Child - Bl (M500-C)	A	12277:14284	94.96	T	612:695	4.67	ND1	Y (Pro)
10	A16170G	Mother - Bu (1267)	A	17593:21027	94.49	G	1060:1174	5.46	CR	
		Child - Bu (1160)	A	15700:21013	99.95	G	4:5	0.025		
		Mother - Bl (SC16)	A	8155:10691	96.19	G	332:413	3.8	CR	
		Child - Bl (SC16-C)	A	10154:13352	99.97	G	1:2	0.013		
11	G9196A	Mother - Bu (839)	G	6061:10535	97.36	A	172:265	2.56	ATP6	N (Asp to Asn)
		Child - Bu (1189)	G	4644:7284	99.81	A	0:6	0.050		
		Mother - Bl (M494)	G	14294:16248	97.83	A	306:362	2.13	ATP6	N (Asp to Asn)
		Child - Bl (M494-C)	G	10236:11242	99.95	A	0:3	0.014		
	T3183C	Mother - Bu (839)	T	12953:24373	99.67	C	26:53	0.211		
		Child - Bu (1189)	T	12210:26317	96.49	C	412:937	3.37	16S	
		Mother - Bl (M494)	T	36453:46743	99.85	C	40:63	0.124		
		Child - Bl (M494-C)	T	30655:39617	96.78	C	970:1333	3.17	16S	
	A15948G	Mother - Bu (839)	A	15680:15099	99.87	G	13:13	0.084		
		Child - Bu (1189)	A	21533:19721	95.35	G	1039:902	4.48	tRNA^thr^	
		Mother - Bl (M494)	A	24430:24443	99.95	G	7:7	0.029		
		Child - Bl (M494-C)	A	30887:30673	96.64	G	1074:1041	3.32	tRNA^thr^	
12	C11288T	Mother - Bu (740)	C	18404:14646	99.97	T	2:1	0.009		
		Child - Bu (718)	C	68908:55901	95.69	T	3140:2418	4.26	ND4	Y (Leu)
		Mother - Bl (M211)	C	38204:36874	99.95	T	8:17	0.033		
		Child - Bl (M211-C)	C	46511:43582	96.58	T	1651:1523	3.4	ND4	Y (Leu)
13	T596C	Mother - Bu (739)	T	3088:856	84.7	C	552:155	15.18	tRNA^phe^	
		Child - Bu (725)	T	9324:2728	99.37	C	3:13	0.132		
		Mother - Bl (M200)	T	1745:1125	95.15	C	93:52	4.8	tRNA^phe^	
		Child - Bl (M200-C)	T	5520:3270	99.82	C	8:4	0.136		
14	A926G	Mother - Bu (605)	A	7528:4119	96.48	G	275:147	3.49	12S	
		Child - Bu (619)	A	23698:18664	99.95	G	7:5	0.028		
		Mother - Bl (M240)	A	3882:3469	96.29	G	149:131	3.66	12S	
		Child - Bl (M240-C)	A	4476:4483	99.92	G	2:2	0.043		
15	A14573G	Mother - Bu (632)	A	3406:2340	70.79	G	1390:966	29.02	ND6	N (Val to Ala)
		Child - Bu (696)	A	5484:4707	99.73	G	1:4	0.049		
		Mother - Bl (M236)	A	7546:6525	77.51	G	2240:1839	22.47	ND6	N (Val to Ala)
		Child - Bl (M236-C)	A	8151:7438	99.95	G	3:1	0.026		
	A214G	Mother - Bu (632)	A	886:2300	99.75	G	3:4	0.219		
		Child - Bu (696)	A	1739:2434	91.43	G	156:230	8.45	CR	
		Mother - Bl (M236)	A	3777:4526	99.99	G	0:1	0.012		
		Child - Bl (M236-C)	A	3426:4146	96.87	G	119:124	3.1	CR	
16	A16240G	Mother - Bu (531)	A	1424:1744	99.75	G	3:2	0.157		
		Child - Bu (572)	A	24590:25415	90.81	G	2527:2455	9.04	CR	
		Mother - Bl (M-188)	A	16073:16853	99.88	G	4:9	0.039		
		Child - Bl (M188-C)	A	13732:14444	94.28	G	823:855	5.61	CR	
17	A9983G	Mother - Bu (616)	A	2944:7014	99.83	G	5:12	0.170		
		Child - Bu (643)	A	11623:20991	97.33	G	314:547	2.56	CO3	Y (Trp)
		Mother - Bl (M252)	A	11354:14962	99.83	G	18:22	0.152		
		Child - Bl (M252-C)	A	17123:22272	97.91	G	346:468	2.02	CO3	Y (Trp)

**Table 3:** Substitution-based error rates for A, C, G, and T base calls. Error Rates represent the total numbers of calls made in error at all nucleotide positions (16,569 positions) divided by the total number of sequencing base calls.

	rCRS>A	rCRS>C	rCRS>G	rCRS>T
Error Rate	0.0343	0.0565	0.0331	0.0304

rCRS: revised Cambridge Reference Sequence.

**Table 4:** Nucleotide positions and adjacent sequence for locations with the highest frequency error for each nucleotide type (A, C, G, and T). The adjacent sequence preceding and following the error site (noted as *) as given by the L-strand of the rCRS (NC_012920). The surrounding sequence is limited to motifs of three nucleotides unless the adjacent sequence is a homopolymeric stretch, in which case the entire complement is given.

rCRS>A error			
Adjacent Sequence	rCRS nt	np	Error
AAA*TAC	C	2785	0.669
TCA*AAG	T	2445	0.722
ATA*AAAA	T	6415	0.732
AAA*AGT	C	2756	0.836
AGA*GAG	C	2718	0.875
AAG*AAC	G	2471	0.900
CAA*ACG	G	2716	0.920
GAA*ACC	G	2724	1.013
AAC*AAC	T	10,304	1.018
AAAAA*AAAAAA	T	8496	1.079
AAC*AAAA	C	8523	1.095
CTA*AAA	G	10,260	1.130
AAG*AAA	G	2449	1.223
CCA*AAAAA	T	8490	1.395
CTA*AAA	C	10,296	1.421
AGT*AAA	T	1115	1.517
CAC*AAA	C	3477	1.668
TAA*ACA	C	2708	2.048
AAC*AAAA	G	8533	2.355
GGT*AAAAAAA	T	2456	2.457
CCA*AAAA	T	3464	2.784
CGG*AAA	C	2479	3.371
AAAAA*TTC	T	2806	3.634
CCCC*AAAA	T	3488	4.629
AAAAAAAG*AAAAGG	T	2465	4.787
rCRS>C error			
Adjacent Sequence	rCRS nt	np	Error
AAC*CTA	A	1142	1.971
TCT*CAC	T	3473	1.996
CTC*CCA	A	14,914	2.010
ATT*CCC	A	2412	2.051
TAG*CCT	G	8573	2.157
ATC*CCG	A	3523	2.214
TCC*CCA	A	297	2.261
AAC*CGG	T	2475	2.363
CCCC*CCCC	A	14,813	2.532
TAA*CCT	A	1104	2.629
CCCCCCC*CCCCC	T	310	2.702
TAC*CCT	A	6355	2.840
TGA*CCC	G	10,290	3.220
CAA*CCCCCCC	A	302	3.259
GAT*CCCC	A	1082	3.365
TCT*CCA	A	3505	3.387
CAA*CCC	A	8512	3.419
ATT*CCT	A	10,239	3.531
CCC*CCC	A	6316	3.635
AAA*CTC	A	3468	4.003
TCC*CCC	A	5208	4.003
CCT*CCC	A	8577	4.318
AGA*CCCC	G	3483	4.614
ACA*CCC	A	3447	4.753
CAA*CCT	T	8567	4.799
TTC*CCA	A	3475	4.954
CCT*CCA	A	10,283	5.115
AAA*CCC	A	3492	5.852
ATT*CCCCC	G	8557	5.990
AAC*CCC	A	5192	6.157
CTA*CCT	A	10,306	6.723
TTTT*CCCC	A	10,277	7.210
AAA*CCCCC	A	6419	10.035
ATC*CCC	A	3511	11.371
CTT*CCCC	A	2487	14.821
rCRS>G error			
Adjacent Sequence	rCRS nt	np	Error
CAC*CCC	T	466	0.301
CAG*GCC	A	3243	0.301
AAC*GGC	T	5717	0.311
GAG*GTT	T	944	0.320
GGGG*AGC	A	16,037	0.325
AAA*CCCCC	A	16,183	0.326
CCC*CCC	A	16,293	0.328
CAG*TTA	T	578	0.329
TGG*GAT	T	2010	0.329
AAC*CGG	T	2475	0.337
CGC*GAC	T	3456	0.347
TTA*CCC	C	10,287	0.358
AGG*GTA	T	1335	0.376
AAG*GCC	A	5539	0.382
AGG*GGC	T	1349	0.406
CCG*ATA	T	7429	0.444
CGG*GCT	T	1180	0.487
GGG*ATA	C	2703	0.501
AAC*GCT	T	1129	0.577
AAC*GGG	T	1071	0.628
AAG*GCC	A	3482	0.672
AGG*CCT	T	2778	0.950
TGG*TTC	T	7480	1.069
AGG*TAT	T	7522	1.183
TGG*GCT	A	2734	2.458
rCRS>T error			
Adjacent Sequence	rCRS nt	np	Error
ATC*CCC	A	16	0.345
CAA*CCCCCCC	A	302	0.343
GTC*CCCCCC	A	432	0.322
ACA*TTA	G	1113	0.320
GAT*AAAA	T	2352	0.834
AAG*TTA	G	2454	0.630
TTT*ATT	A	2740	1.058
TTT*TTA	A	2745	0.679
ATT*ATG	A	2748	0.570
TAT*CCC	A	4455	0.347
CTA*TAC	C	5107	0.360
TCT*CCT	A	5347	0.348
ATC*CCT	A	7649	0.346
TCT*TTC	G	8541	0.509
TCG*TTC	C	8546	0.579
TTC*TTC	A	8550	0.382
AGC*GGC	G	8856	0.410
ACC*CCT	A	9425	0.333
CAG*CAC	C	11,635	0.377
CCCCCC*CTA	A	11,873	0.354
ACC*CCC	A	12,400	0.331
GCT*CCT	A	14,988	0.370
ATC*CCT	A	15,401	0.426
TCC*CCC	A	15,408	0.579
CCC*CCC	A	16,293	0.341

## Supplementary Materials

The following are available online at http://www.mdpi.com/2073-4425/9/3/124/s1. **Table S1**: Haplotypes, haplogroups, and heteroplasmy for each of the 39 mother-child pairs. Heteroplasmy observed in buccal cell (Bu) and blood (Bl) samples. The frequency of shared heteroplasmy and sites of potential differentiating heteroplasmy are annotated as percentages (%), **Table S2**: Sites of shared, differentiating, and random heteroplasmy for 29 of the 39 pairs; nine of the remaining 10 pairs exhibited no heteroplasmy, while the tenth pair had a shared, nine bp insertion. Read coverage and distribution, gene annotation and protein coding changes are provided. Sites of differentiating heteroplasmy in both buccal and blood cells are highlighted in **BOLD** text. A total of 16 of the 21 differentiating sites are in the coding region of the mtgenome, **Table S3**: Comparison of samples with differentiating heteroplasmy, shared heteroplasmy and random sites of heteroplasmy that are neither shared nor differentiating. No duplicate sites were observed within the datasets for differentiating and shared heteroplasmy, with one site (16093) observed in both the differentiating and shared datasets. A total of 28 of the 96 differentiating and shared sites (29%, buccal and blood combined) have frequencies above 10%. Each range of heteroplasmy is reported from blood (Bl) to buccal (Bu), not lowest to highest value.
