# Supplementary material for: Deep-Coverage MPS Analysis of Heteroplasmic Variants within the mtGenome Allows for Frequent Differentiation of Maternal Relatives
Source: Genes (Basel). 2018 Feb 26;9(3):124. doi: 10.3390/genes9030124 (PMC5867845; doi:10.3390/genes9030124)
Supplement: Supplementary file 1 [file genes-09-00124-s001.zip › Supplemental-final/Table S1.docx]

Supplemental Table 1: Haplotypes, haplogroups, and heteroplasmy for each of the 39 mother-child pairs. Heteroplasmy observed in buccal cell (Bu) and blood (Bl) samples. The frequency of shared heteroplasmy and sites of potential differentiating heteroplasmy are annotated as percentages (%).

| **Mother-Child Pair** | **Sample Number** | **Shared Heteroplasmy** | **Potential Differentiating Heteroplasmy** | **Haplotype** | **Haplogroup** | **Haplotype Differences** |
| --- | --- | --- | --- | --- | --- | --- |
|  |  |  |  |  |  |  |
| 1 | Mother - Bu (807) | A16183G (7.32%) | G185G (2.89%) & T16092T (2.40%) | 73G 146C 185A 228A 263G 295T 315.1C 462T 489C 750G 1438G 2706G 3010A 4216C 4769G 6464A 6554T 7028T 8860G 10398G 11251G 11719A 12127A 12612G 13681G 13708A 14325C 14766T 14798C 15326G 15452A 15496G 16069T 16092C 16126C 16261T | J1c7a | NA |
|  | Child - Bu (803) | A16183G (6.89%) |  | 73G 146C 185A 228A 263G 295T 315.1C 462T 489C 750G 1438G 2706G 3010A 4216C 4769G 6464A 6554T 7028T 8860G 10398G 11251G 11719A 12127A 12612G 13681G 13708A 14325C 14766T 14798C 15326G 15452A 15496G 16069T 16092C 16126C 16261T |  | NA |
|  | Mother - Bl (M490) | A16183G (2.81%) |  | 73G 146C 185A 228A 263G 295T 315.1C 462T 489C 750G 1438G 2706G 3010A 4216C 4769G 6464A 6554T 7028T 8860G 10398G 11251G 11719A 12127A 12612G 13681G 13708A 14325C 14766T 14798C 15326G 15452A 15496G 16069T 16092C 16126C 16261T |  | NA |
|  | Child - Bl (M490-C) | A16183G (2.46%) |  | 73G 146C 185A 228A 263G 295T 315.1C 462T 489C 750G 1438G 2706G 3010A 4216C 4769G 6464A 6554T 7028T 8860G 10398G 11251G 11719A 12127A 12612G 13681G 13708A 14325C 14766T 14798C 15326G 15452A 15496G 16069T 16092C 16126C 16261T |  | NA |
|  |  |  |  |  |  |  |
| 2 | Mother - Bu (618) | T16189C (7.74%) | T16093T (11.52%) | 73G 263G 309.1C 315.1C 497T 524.1A 524.2C 723C 750G 1189C 1438G 1811G 2706G 3395G 3480G 4769G 7028T 8860G 9055A 9698C 10398G 10550G 11071T 11299C 11467G 11485C 11719A 12308G 12372A 14167T 14766T 14798C 15326G 15355A 16093C 16224C 16311C 16519C | K1a4d | NA |
|  | Child - Bu (606) | T16189C (11.07%) | T13581C (5.38%) | 73G 263G 309.1C 315.1C 497T 524.1A 524.2C 723C 750G 1189C 1438G 1811G 2706G 3395G 3480G 4769G 7028T 8860G 9055A 9698C 10398G 10550G 11071T 11299C 11467G 11485C 11719A 12308G 12372A 14167T 14766T 14798C 15326G 15355A 16093C 16224C 16311C 16519C |  | NA |
|  | Mother - Bl (M249) | T16189C (2.81%) |  | 73G 263G 309.1C 315.1C 497T 524.1A 524.2C 723C 750G 1189C 1438G 1811G 2706G 3395G 3480G 4769G 7028T 8860G 9055A 9698C 10398G 10550G 11071T 11299C 11467G 11485C 11719A 12308G 12372A 14167T 14766T 14798C 15326G 15355A 16093C 16224C 16311C 16519C |  | NA |
|  | Child - Bl (M249-C) | T16189C (9.92%) |  | 73G 263G 309.1C 315.1C 497T 524.1A 524.2C 723C 750G 1189C 1438G 1811G 2706G 3395G 3480G 4769G 7028T 8860G 9055A 9698C 10398G 10550G 11071T 11299C 11467G 11485C 11719A 12308G 12372A 14167T 14766T 14798C 15326G 15355A 16093C 16224C 16311C 16519C |  | NA |
|  |  |  |  |  |  |  |
| 3 | Mother - Bu (693) |  | T2746C (20.11%) | 263G 315.1C 750G 1438G 3915A 4769G 8860G 8895A 15326G 16129A 16311C 16519C | H17b | NA |
|  | Child - Bu (677) |  |  | 263G 315.1C 750G 1438G 3915A 4769G 8860G 8895A 15326G 16129A 16311C 16519C |  | NA |
|  | Mother - Bl (M207) |  | T2746C (19.62%) | 263G 315.1C 750G 1438G 3915A 4769G 8860G 8895A 15326G 16129A 16311C 16519C |  | NA |
|  | Child - Bl (M207-C) |  |  | 263G 315.1C 750G 1438G 3915A 4769G 8860G 8895A 15326G 16129A 16311C 16519C |  | NA |
|  |  |  |  |  |  |  |
| 4 | Mother - Bu (704) | T6152C (7.23%) |  | 73G 185A 228A 263G 295T 309.1C 315.1C 462T 489C 750G 1438G 1811G 2706G 3010A 4216C 4769G 7028T 8860G 10398G 11251G 11719A 12612G 13708A 13934T 14766T 14798C 15326G 15452A 16069T 16126C | J1c3/J1c3d | NA |
|  | Child - Bu (630) | T6152C (16.37%) |  | 73G 185A 228A 263G 295T 309.1C 315.1C 462T 489C 750G 1438G 1811G 2706G 3010A 4216C 4769G 7028T 8860G 10398G 11251G 11719A 12612G 13708A 13934T 14766T 14798C 15326G 15452A 16069T 16126C |  | NA |
|  | Mother - Bl (M234) | T6152C (5.04%) |  | 73G 185A 228A 263G 295T 309.1C 315.1C 462T 489C 750G 1438G 1811G 2706G 3010A 4216C 4769G 7028T 8860G 10398G 11251G 11719A 12612G 13708A 13934T 14766T 14798C 15326G 15452A 16069T 16126C |  | NA |
|  | Child - Bl (M234-C) | T6152C (16.48%) |  | 73G 185A 228A 263G 295T 309.1C 315.1C 462T 489C 750G 1438G 1811G 2706G 3010A 4216C 4769G 7028T 8860G 10398G 11251G 11719A 12612G 13708A 13934T 14766T 14798C 15326G 15452A 16069T 16126C |  | NA |
|  |  |  |  |  |  |  |
| 5 | Mother - Bu (406) |  | C16320T (27.57%) | 195C 263G 309.1C 315.1C 750G 961G 1438G 4769G 8448C 8860G 13759A 15326G 16311C | H11a | NA |
|  | Child - Bu (444) |  |  | 195C 263G 309.1C 315.1C 750G 961G 1438G 4769G 8448C 8860G 13759A 15326G 16311C |  | NA |
|  | Mother - Bl (M137) |  | C16320T (5.07%) | 195C 263G 309.1C 315.1C 750G 961G 1438G 4769G 8448C 8860G 13759A 15326G 16311C |  | NA |
|  | Child - Bl (M137-C) |  |  | 195C 263G 309.1C 315.1C 750G 961G 1438G 4769G 8448C 8860G 13759A 15326G 16311C |  | NA |
|  |  |  |  |  |  |  |
| 6 | Mother - Bu (762) | T10873C (2.53%) |  | 73G 263G 315.1C 750G 1438G 2706G 3197C 4553C 4769G 7028T 8860G 9477A 9667G 11467G 11719A 12308G 12372A 13617C 14766T 14793G 15218G 15326G 16192T 16256T 16270T 16291T 16399G | U5a1b1a/U5a1b1a2 | NA |
|  | Child - Bu (702) | T10873C (6.66%) |  | 73G 263G 315.1C 750G 1438G 2706G 3197C 4553C 4769G 7028T 8860G 9477A 9667G 11467G 11719A 12308G 12372A 13617C 14766T 14793G 15218G 15326G 16192T 16256T 16270T 16291T 16399G |  | NA |
|  | Mother - Bl (M210) |  | G11825A (2.15%) | 73G 263G 315.1C 750G 1438G 2706G 3197C 4553C 4769G 7028T 8860G 9477A 9667G 11467G 11719A 12308G 12372A 13617C 14766T 14793G 15218G 15326G 16192T 16256T 16270T 16291T 16399G |  | NA |
|  | Child - Bl (M210-C) | T10873C (5.40%) |  | 73G 263G 315.1C 750G 1438G 2706G 3197C 4553C 4769G 7028T 8860G 9477A 9667G 11467G 11719A 12308G 12372A 13617C 14766T 14793G 15218G 15326G 16192T 16256T 16270T 16291T 16399G |  | NA |
|  |  |  |  |  |  |  |
| 7 | Mother - Bu (1134) |  | T9179C (14.93%) & T16189C (2.58%) | 263G 309.1C 309.2C 315.1C 316A 750G 1438G 1821G 2206A 3010A 4733C 4767G 4769G 8450C 8860G 13708A 14968C 15326G 16213A 16291T 16519C | H1j2a | NA |
|  | Child - Bu (1099) |  | C10980T (4.44%) | 263G 309.1C 309.2C 315.1C 316A 750G 1438G 1821G 2206A 3010A 4733C 4767G 4769G 8450C 8860G 13708A 14968C 15326G 16213A 16291T 16519C |  | NA |
|  | Mother - Bl (M502G) |  | T9179C (12.77%) | 263G 309.1C 309.2C 315.1C 316A 750G 1438G 1821G 2206A 3010A 4733C 4767G 4769G 8450C 8860G 13708A 14968C 15326G 16213A 16291T 16519C |  | NA |
|  | Child - Bl (M501) |  |  | 263G 309.1C 309.2C 315.1C 316A 750G 1438G 1821G 2206A 3010A 4733C 4767G 4769G 8450C 8860G 13708A 14968C 15326G 16213A 16291T 16519C |  | NA |
|  |  |  |  |  |  |  |
| 8 | Mother - Bu (403) |  |  | 152C 263G 315.1C 750G 1438G 4769G 8592A 8860G 10394T 15326G 16519C | H16a/H16a1 | NA |
|  | Child - Bu (459) |  |  | 152C 263G 315.1C 750G 1438G 4769G 8592A 8860G 10394T 15326G 16519C |  | NA |
|  | Mother - Bl (M117) |  |  | 152C 263G 315.1C 750G 1438G 4769G 8592A 8860G 10394T 15326G 16519C |  | NA |
|  | Child - Bl (M117-C) |  |  | 152C 263G 315.1C 750G 1438G 4769G 8592A 8860G 10394T 15326G 16519C |  | NA |
|  |  |  |  |  |  |  |
| 9 | Mother - Bu (875) |  | C1948T (2.06%) | 263G 309.1C 315.1C 456T 750G 1438G 4769G 6872G 8860G 15326G 16256T 16304C | H5 | NA |
|  | Child - Bu (825) |  |  | 263G 309.1C 315.1C 456T 750G 1438G 4769G 6872G 8860G 15326G 16256T 16304C |  | NA |
|  | Mother - Bl (M493) |  |  | 263G 309.1C 315.1C 456T 750G 1438G 4769G 6872G 8860G 15326G 16256T 16304C |  | NA |
|  | Child - Bl (M493-C) |  |  | 263G 309.1C 315.1C 456T 750G 1438G 4769G 6872G 8860G 15326G 16256T 16304C |  | NA |
|  |  |  |  |  |  |  |
| 10 | Mother - Bu (659) |  | G14040A (7.86%) | 263G 315.1C 750G 1438G 3381G 4769G 8860G 8888C 9966A 14470A 15326G 16148T 16519C | H10h | NA |
|  | Child - Bu (722) |  |  | 263G 315.1C 750G 1438G 3381G 4769G 8860G 8888C 9966A 14470A 15326G 16148T 16519C |  | NA |
|  | Mother - Bl (M242) |  | G14040A (5.89%) | 263G 315.1C 750G 1438G 3381G 4769G 8860G 8888C 9966A 14470A 15326G 16148T 16519C |  | NA |
|  | Child - Bl (M242-C) |  |  | 263G 315.1C 750G 1438G 3381G 4769G 8860G 8888C 9966A 14470A 15326G 16148T 16519C |  | NA |
|  |  |  |  |  |  |  |
| 11 | Mother - Bu (411) |  | T14461C (2.87%) | 73G 185A 228A 263G 295T 309.1C 315.1C 462T 489C 523del 524del 750G 1438G 2706G 3010A 4216C 4688C 4769G 5198G 5978G 7028T 7340A 7888T 8860G 10398G 11251G 11719A 12612G 13434G 13708A 14766T 14798C 15326G 15452A 16069T 16126C | J1c5d | NA |
|  | Child - Bu (401) |  |  | 73G 185A 228A 263G 295T 309.1C 315.1C 462T 489C 523del 524del 750G 1438G 2706G 3010A 4216C 4688C 4769G 5198G 5978G 7028T 7340A 7888T 8860G 10398G 11251G 11719A 12612G 13434G 13708A 14766T 14798C 15326G 15452A 16069T 16126C |  | NA |
|  | Mother - Bl (M132) |  | T14461C (2.41%) | 73G 185A 228A 263G 295T 309.1C 315.1C 462T 489C 523del 524del 750G 1438G 2706G 3010A 4216C 4688C 4769G 5198G 5978G 7028T 7340A 7888T 8860G 10398G 11251G 11719A 12612G 13434G 13708A 14766T 14798C 15326G 15452A 16069T 16126C |  | NA |
|  | Child - Bl (M132-C) |  |  | 73G 185A 228A 263G 295T 309.1C 315.1C 462T 489C 523del 524del 750G 1438G 2706G 3010A 4216C 4688C 4769G 5198G 5978G 7028T 7340A 7888T 8860G 10398G 11251G 11719A 12612G 13434G 13708A 14766T 14798C 15326G 15452A 16069T 16126C |  | NA |
|  |  |  |  |  |  |  |
| 12 | Mother - Bu (665) |  |  | 73G 263G 309.1C 315.1C 524.1A 524.2C 709A 750G 1438G 1888A 2706G 3349G 4216C 4769G 4917G 7028T 7444A 8697A 8860G 10463C 11251G 11719A 12633A 12930G 13368A 14766T 14905A 15326G 15452A 15607G 15928A 16126C 16186T 16189C 16294T 16519C | T1a | NA |
|  | Child - Bu (746) |  |  | 73G 263G 309.1C 315.1C 524.1A 524.2C 709A 750G 1438G 1888A 2706G 3349G 4216C 4769G 4917G 7028T 7444A 8697A 8860G 10463C 11251G 11719A 12633A 12930G 13368A 14766T 14905A 15326G 15452A 15607G 15928A 16126C 16186T 16189C 16294T 16519C |  | NA |
|  | Mother - Bl (M204) |  |  | 73G 263G 309.1C 315.1C 524.1A 524.2C 709A 750G 1438G 1888A 2706G 3349G 4216C 4769G 4917G 7028T 7444A 8697A 8860G 10463C 11251G 11719A 12633A 12930G 13368A 14766T 14905A 15326G 15452A 15607G 15928A 16126C 16186T 16189C 16294T 16519C |  | NA |
|  | Child - Bl (M204-C) |  |  | 73G 263G 309.1C 315.1C 524.1A 524.2C 709A 750G 1438G 1888A 2706G 3349G 4216C 4769G 4917G 7028T 7444A 8697A 8860G 10463C 11251G 11719A 12633A 12930G 13368A 14766T 14905A 15326G 15452A 15607G 15928A 16126C 16186T 16189C 16294T 16519C |  | NA |
|  |  |  |  |  |  |  |
| 13 | Mother - Bu (711) |  | G11825A (6.54%) | 146C 263G (309.1C 309.2C 315.1C) 750G 1438G 3342T 3394C 4769G 8860G 8940T 9383T 14573G 15326G 16519C | H35 | 309.1C 309.2C 315.1C below coverage threshold of 200 reads |
|  | Child - Bu (737) |  | T11616C (5.93%) & T12375C (27.82%) | 146C 263G **309.1C 309.2C** **315.1C** 750G 1438G 3342T 3394C 4769G 8860G 8940T 9383T 14573G 15326G 16519C |  | NA |
|  | Mother - Bl (M203) |  | G11825A (2.74%) | 146C 263G (309.1C 309.2C 315.1C) 750G 1438G 3342T 3394C 4769G 8860G 8940T 9383T 14573G 15326G 16519C |  | 309.1C 309.2C 315.1C below coverage threshold of 200 reads |
|  | Child - Bl (M203-C) |  | G9907A (2.20%) & T12375C (23.95%) | 146C 263G **309.1C 309.2C** **315.1C** 750G 1438G 3342T 3394C 4769G 8860G 8940T 9383T 14573G 15326G 16519C |  | NA |
|  |  |  |  |  |  |  |
| 14 | Mother - Bu (729) | A1656A (2.11%) | T16093T (4.12%) | 73G 143A 153G 195C 225A 263G 315.1C 750G 1438G 1656del 1719A 2706G 4769G 6221C 6371T 7028T 8860G 9840A 11719A 12246T 12705T 13035A 13656C 13966G 14470C 14766T 15326G 16093C 16189C 16223T 16278T 16519C | X2o | NA |
|  | Child - Bu (684) | A1656A (2.52%) | A13790G (11.22%) | 73G 143A 153G 195C 225A 263G 315.1C 750G 1438G 1656del 1719A 2706G 4769G 6221C 6371T 7028T 8860G 9840A 11719A 12246T 12705T 13035A 13656C 13966G 14470C 14766T 15326G 16093C 16189C 16223T 16278T 16519C |  | NA |
|  | Mother - Bl (M213) | A1656A (2.77%) |  | 73G 143A 153G 195C 225A 263G 315.1C 750G 1438G 1656del 1719A 2706G 4769G 6221C 6371T 7028T 8860G 9840A 11719A 12246T 12705T 13035A 13656C 13966G 14470C 14766T 15326G 16093C 16189C 16223T 16278T 16519C |  | NA |
|  | Child - Bl (M213-C) | A1656A (2.68%) | A11362G (2.11%) & A13790G (11.10%) | 73G 143A 153G 195C 225A 263G 315.1C 750G 1438G 1656del 1719A 2706G 4769G 6221C 6371T 7028T 8860G 9840A 11719A 12246T 12705T 13035A 13656C 13966G 14470C 14766T 15326G 16093C 16189C 16223T 16278T 16519C |  | NA |
|  |  |  |  |  |  |  |
| 15 | Mother - Bu (1091) | A3243G (30.72%), A5539A (41.94%) & C16192C (19.23%) |  | 73G 263G 309.1C 315.1C 524.1A 524.2C 750G 1438G 2706G 3197C 4769G **5539G** 7028T 7269A 8860G 9477A 9548A 11467G 11719A 12308G 12372A 13617C 14766T 14793G 15301A 15326G 16189C 16192T 16256T 16270T 16526A | U5a2b/U5a2b4 | 3243G (30.72%) &16191.C (42.87%) |
|  | Child - Bu (1111) | A3243A (33.10%), A5539G (24.54%) & C16192C (14.10%) | A16183C (2.15%) | 73G 263G 309.1C 315.1C 524.1A 524.2C 750G 1438G 2706G 3197C **3243G** 4769G 7028T 7269A 8860G 9477A 9548A 11467G 11719A 12308G 12372A 13617C 14766T 14793G 15301A 15326G 16189C **16191.1C** 16192T 16256T 16270T 16526A |  | 5539 (24.54%) |
|  | Mother - Bl (M512) | A3243G (13.13%), A5539A (23.13%) & C16192C (22.78%) |  | 73G 263G 309.1C 315.1C 524.1A 524.2C 750G 1438G 2706G 3197C 4769G **5539G** 7028T 7269A 8860G 9477A 9548A 11467G 11719A 12308G 12372A 13617C 14766T 14793G 15301A 15326G 16189C 16192T 16256T 16270T 16526A |  | 3243G (13.13%) &16191.C (32.40%) |
|  | Child - Bl (M512-C) | A3243A (41.01%), A5539G (31.26%) & C16192C (17.30%) |  | 73G 263G 309.1C 315.1C 524.1A 524.2C 750G 1438G 2706G 3197C **3243G** 4769G 7028T 7269A 8860G 9477A 9548A 11467G 11719A 12308G 12372A 13617C 14766T 14793G 15301A 15326G 16189C **16191.1C** 16192T 16256T 16270T 16526A |  | 5539 (31.26%) |
|  |  |  |  |  |  |  |
| 16 | Mother - Bu (1098) | T16093C (11.53%) | A200A (3.26%) | 200G 263G **309.1C 315.1C** 523del 524del 750G 1438G 3462T 4107T 4769G 7403G 8860G 11440A 11485C 14872T 15326G 16261T 16291T 16311C 16362C 16519C | H13b1b | 16093C (11.53%) |
|  | Child - Bu (1100) | T16093T (3.45%) |  | 200G 263G **309.1C 315.1C** 523del 524del 750G 1438G 3462T 4107T 4769G 7403G 8860G 11440A 11485C 14872T 15326G **16093C** 16261T 16291T 16311C 16362C 16519C |  | NA |
|  | Mother - Bl (M520) | T16093C (9.12%) | A200A (2.26%) | 200G 263G 523del 524del 750G 1438G 3462T 4107T 4769G 7403G 8860G 11440A 11485C 14872T 15326G 16261T 16291T 16311C 16362C 16519C |  | 309.1C 315.1C below coverage threshold of 200 reads & 16093 (9.12%) |
|  | Child - Bl (M520-C) |  |  | 200G 263G **309.1C 315.1C** 523del 524del 750G 1438G 3462T 4107T 4769G 7403G 8860G 11440A 11485C 14872T 15326G **16093C** 16261T 16291T 16311C 16362C 16519C |  | NA |
|  |  |  |  |  |  |  |
| 17 | Mother - Bu (795) |  |  | 73G 263G 315.1C 750G 1438G 2706G 3197C 4769G 7028T 8860G 9477A 9667G 11467G 11719A 12308G 12372A 13617C 14766T 14793G 15218G 15326G 16192T 16256T 16270T 16362C 16399G | U5a1b | NA |
|  | Child - Bu (843) |  |  | 73G 263G 315.1C 750G 1438G 2706G 3197C 4769G 7028T 8860G 9477A 9667G 11467G 11719A 12308G 12372A 13617C 14766T 14793G 15218G 15326G 16192T 16256T 16270T 16362C 16399G |  | NA |
|  | Mother - Bl (M478) |  |  | 73G 263G 315.1C 750G 1438G 2706G 3197C 4769G 7028T 8860G 9477A 9667G 11467G 11719A 12308G 12372A 13617C 14766T 14793G 15218G 15326G 16192T 16256T 16270T 16362C 16399G |  | NA |
|  | Child - Bl (M478-C) |  |  | 73G 263G 315.1C 750G 1438G 2706G 3197C 4769G 7028T 8860G 9477A 9667G 11467G 11719A 12308G 12372A 13617C 14766T 14793G 15218G 15326G 16192T 16256T 16270T 16362C 16399G |  | NA |
|  |  |  |  |  |  |  |
| 18 | Mother - Bu (800) |  |  | 73G 146C 242T 263G 295T 315.1C 462T 489C 750G 1438G 2158C 2706G 3010A 4216C 4769G 5460A 7028T 8269A 8557A 8860G 10398G 11251G 11719A 12007A 12612G 13708A 13879C 14766T 15326G 15452A 16069T 16126C 16145A 16172C 16222T 16261T 16311C | J1b1a1 | NA |
|  | Child - Bu (871) |  | A215G (3.75%) | 73G 146C 242T 263G 295T 315.1C 462T 489C 750G 1438G 2158C 2706G 3010A 4216C 4769G 5460A 7028T 8269A 8557A 8860G 10398G 11251G 11719A 12007A 12612G 13708A 13879C 14766T 15326G 15452A 16069T 16126C 16145A 16172C 16222T 16261T 16311C |  | NA |
|  | Mother - Bl (M480) |  |  | 73G 146C 242T 263G 295T 315.1C 462T 489C 750G 1438G 2158C 2706G 3010A 4216C 4769G 5460A 7028T 8269A 8557A 8860G 10398G 11251G 11719A 12007A 12612G 13708A 13879C 14766T 15326G 15452A 16069T 16126C 16145A 16172C 16222T 16261T 16311C |  | NA |
|  | Child - Bl (M480-C) |  |  | 73G 146C 242T 263G 295T 315.1C 462T 489C 750G 1438G 2158C 2706G 3010A 4216C 4769G 5460A 7028T 8269A 8557A 8860G 10398G 11251G 11719A 12007A 12612G 13708A 13879C 14766T 15326G 15452A 16069T 16126C 16145A 16172C 16222T 16261T 16311C |  | NA |
|  |  |  |  |  |  |  |
| 19 | Mother - Bu (1122) |  | A214G (2.47%) & T310C (7.34%) | 73G 150T 263G 309.1C 315.1C 750G 896G 1438G 1721T 2581G 2706G 3197C 4732G 4769G 7028T 7768G 8860G 9386C 9477A 11467G 11719A 12308G 12372A 12406A 13617C 13637G 14182C 14766T 15326G 15511C 16192T 16311C | U5b2a1a1a | NA |
|  | Child - Bu (1119) |  | A4191T (4.17%) | 73G 150T 263G 309.1C 315.1C 750G 896G 1438G 1721T 2581G 2706G 3197C 4732G 4769G 7028T 7768G 8860G 9386C 9477A 11467G 11719A 12308G 12372A 12406A 13617C 13637G 14182C 14766T 15326G 15511C 16192T 16311C |  | NA |
|  | Mother - Bl (M500) |  |  | 73G 150T 263G 309.1C 315.1C 750G 896G 1438G 1721T 2581G 2706G 3197C 4732G 4769G 7028T 7768G 8860G 9386C 9477A 11467G 11719A 12308G 12372A 12406A 13617C 13637G 14182C 14766T 15326G 15511C 16192T 16311C |  | NA |
|  | Child - Bl (M500-C) |  | A4191T (4.67%) | 73G 150T 263G 309.1C 315.1C 750G 896G 1438G 1721T 2581G 2706G 3197C 4732G 4769G 7028T 7768G 8860G 9386C 9477A 11467G 11719A 12308G 12372A 12406A 13617C 13637G 14182C 14766T 15326G 15511C 16192T 16311C |  | NA |
|  |  |  |  |  |  |  |
| 20 | Mother - Bu (1267) | T2352T (48.11%) | T310C (7.88%), G11149A (2.16%) & A16170G (5.46%) | 73G 185A 228A 263G 295T 315.1C 462T 489C 750G 1438G 2352C 2387C 2706G 3010A 4216C 4769G 5198G 7028T 8860G 10192T 10398G 10598G 11251G 11719A 12612G 13708A 14766T 14798C 15326G 15452A 16069T 16126C | J1c5a1 | NA |
|  | Child - Bu (1160) | T2352T (26.81%) |  | 73G 185A 228A 263G 295T 315.1C 462T 489C 750G 1438G 2352C 2387C 2706G 3010A 4216C 4769G 5198G 7028T 8860G 10192T 10398G 10598G 11251G 11719A 12612G 13708A 14766T 14798C 15326G 15452A 16069T 16126C |  | NA |
|  | Mother - Bl (SC16) | T2352T (47.93%) | A16170G (5.46%) | 73G 185A 228A 263G 295T 315.1C 462T 489C 750G 1438G 2352C 2387C 2706G 3010A 4216C 4769G 5198G 7028T 8860G 10192T 10398G 10598G 11251G 11719A 12612G 13708A 14766T 14798C 15326G 15452A 16069T 16126C |  | NA |
|  | Child - Bl (SC16-C) | T2352T (26.84%) |  | 73G 185A 228A 263G 295T 315.1C 462T 489C 750G 1438G 2352C 2387C 2706G 3010A 4216C 4769G 5198G 7028T 8860G 10192T 10398G 10598G 11251G 11719A 12612G 13708A 14766T 14798C 15326G 15452A 16069T 16126C |  | NA |
|  |  |  |  |  |  |  |
| 21 | Mother - Bu (508) |  |  | 73G 150T 263G 309.1C 315.1C 524.1A 524.2C 750G 1438G 1721T 1834C 2706G 3197C 4732G 4769G 5452T 7028T 7768G 8705C 8860G 9477A 11467G 11719A 12308G 12372A 13617C 13637G 14182C 14766T 15326G 15511C 15924G 16519C | U5b2a1a2 | NA |
|  | Child - Bu (544) |  |  | 73G 150T 263G 309.1C 315.1C 524.1A 524.2C 750G 1438G 1721T 1834C 2706G 3197C 4732G 4769G 5452T 7028T 7768G 8705C 8860G 9477A 11467G 11719A 12308G 12372A 13617C 13637G 14182C 14766T 15326G 15511C 15924G 16519C |  | NA |
|  | Mother - Bl (M196) |  | T16172C (4.07%) | 73G 150T 263G 309.1C 315.1C 524.1A 524.2C 750G 1438G 1721T 1834C 2706G 3197C 4732G 4769G 5452T 7028T 7768G 8705C 8860G 9477A 11467G 11719A 12308G 12372A 13617C 13637G 14182C 14766T 15326G 15511C 15924G 16519C |  | NA |
|  | Child - Bl (M196-C) |  |  | 73G 150T 263G 309.1C 315.1C 524.1A 524.2C 750G 1438G 1721T 1834C 2706G 3197C 4732G 4769G 5452T 7028T 7768G 8705C 8860G 9477A 11467G 11719A 12308G 12372A 13617C 13637G 14182C 14766T 15326G 15511C 15924G 16519C |  | NA |
|  |  |  |  |  |  |  |
| 22 | Mother - Bu (589) |  | T310C (5.36%) | 263G 315.1C 750G 1438G 3010A 4769G 6249A 8602C 8860G 14212C 15326G 16189C 16519C | H1g1 | NA |
|  | Child - Bu (491) |  |  | 263G 315.1C 750G 1438G 3010A 4769G 6249A 8602C 8860G 14212C 15326G 16189C 16519C |  | NA |
|  | Mother - Bl (M186) |  |  | 263G 315.1C 750G 1438G 3010A 4769G 6249A 8602C 8860G 14212C 15326G 16189C 16519C |  | NA |
|  | Child - Bl (M186-C) |  |  | 263G 315.1C 750G 1438G 3010A 4769G 6249A 8602C 8860G 14212C 15326G 16189C 16519C |  | NA |
|  |  |  |  |  |  |  |
| 23 | Mother - Bu (839) | C11635T (8.34%) | T195C (6.72%), T310C (8.56%) & G9196A (2.56%) | 263G 315.1C 456T 480C 523del 524del 750G 1438G 4336C 4769G 8767G 8860G 15326G 15833T 16304C | H5a1 | NA |
|  | Child - Bu (1189) | C11635T (17.93%) | T3183C (3.37%) & A15948G (4.48%) | 263G 315.1C 456T 480C 523del 524del 750G 1438G 4336C 4769G 8767G 8860G 15326G 15833T 16304C |  | NA |
|  | Mother - Bl (M494) | C11635T (7.23%) | G9196A (2.13%) | 263G 315.1C 456T 480C 523del 524del 750G 1438G 4336C 4769G 8767G 8860G 15326G 15833T 16304C |  | NA |
|  | Child - Bl (M494-C) | C11635T (19.88%) | T3183C (3.17%) & A15948G (3.32%) | 263G 315.1C 456T 480C 523del 524del 750G 1438G 4336C 4769G 8767G 8860G 15326G 15833T 16304C |  | NA |
|  |  |  |  |  |  |  |
| 24 | Mother - Bu (740) |  |  | 73G 263G 315.1C 750G 1438G 3010A 4769G 5460A 5899.1C 8860G 15326G 15817G 16519C | H1e2c | NA |
|  | Child - Bu (718) |  | C11288T (4.26%) | 73G 263G 315.1C 750G 1438G 3010A 4769G 5460A 5899.1C 8860G 15326G 15817G 16519C |  | NA |
|  | Mother - Bl (M211) |  |  | 73G 263G 315.1C 750G 1438G 3010A 4769G 5460A 5899.1C 8860G 15326G 15817G 16519C |  | NA |
|  | Child - Bl (M211-C) |  | C11288T (3.40%) | 73G 263G 315.1C 750G 1438G 3010A 4769G 5460A 5899.1C 8860G 15326G 15817G 16519C |  | NA |
|  |  |  |  |  |  |  |
| 25 | Mother - Bu (489) |  |  | 263G 315.1C 750G 1438G 3381G 4769G 8860G 8888C 9966A 14470A 15326G 16519C | H10h | NA |
|  | Child - Bu (522) |  |  | 263G 315.1C 750G 1438G 3381G 4769G 8860G 8888C 9966A 14470A 15326G 16519C |  | NA |
|  | Mother - Bl (M195) |  |  | 263G 315.1C 750G 1438G 3381G 4769G 8860G 8888C 9966A 14470A 15326G 16519C |  | NA |
|  | Child - Bl (M195-C) |  |  | 263G 315.1C 750G 1438G 3381G 4769G 8860G 8888C 9966A 14470A 15326G 16519C |  | NA |
|  |  |  |  |  |  |  |
| 26 | Mother - Bu (799) |  |  | 73G 150T 263G 309.1C 315.1C 455del 750G 1438G 2706G 3197C 4769G 7028T 8860G 9477A 9548A 11467G 11719A 12308G 12372A 13351T 13617C 14684T 14766T 14793G 15326G 16168T 16192T 16256T 16270T 16304C 16526A | U5a2b3a | NA |
|  | Child - Bu (848) |  |  | 73G 150T 263G 309.1C 315.1C 455del 750G 1438G 2706G 3197C 4769G 7028T 8860G 9477A 9548A 11467G 11719A 12308G 12372A 13351T 13617C 14684T 14766T 14793G 15326G 16168T 16192T 16256T 16270T 16304C 16526A |  | NA |
|  | Mother - Bl (M477) |  |  | 73G 150T 263G 309.1C 315.1C 455del 750G 1438G 2706G 3197C 4769G 7028T 8860G 9477A 9548A 11467G 11719A 12308G 12372A 13351T 13617C 14684T 14766T 14793G 15326G 16168T 16192T 16256T 16270T 16304C 16526A |  | NA |
|  | Child - Bl (M477-C) |  |  | 73G 150T 263G 309.1C 315.1C 455del 750G 1438G 2706G 3197C 4769G 7028T 8860G 9477A 9548A 11467G 11719A 12308G 12372A 13351T 13617C 14684T 14766T 14793G 15326G 16168T 16192T 16256T 16270T 16304C 16526A |  | NA |
|  |  |  |  |  |  |  |
| 27 | Mother - Bu (681) |  |  | 73G 150T 152C 195C 215G 263G 295T 310.1T 315.1C 319C 489C 513A 750G 1438G 1850C 2706G 4216C 4375T 4769G 7028T 7476T 7789A 8860G 10398G 10499G 11251G 11377A 11719A 12612G 13708A 13722G 13830C 14133G 14766T 15257A 15326G 15452A 16069T 16126C 16145A 16231C 16261T | J2a1a1a | NA |
|  | Child - Bu (686) |  |  | 73G 150T 152C 195C 215G 263G 295T 310.1T 315.1C 319C 489C 513A 750G 1438G 1850C 2706G 4216C 4375T 4769G 7028T 7476T 7789A 8860G 10398G 10499G 11251G 11377A 11719A 12612G 13708A 13722G 13830C 14133G 14766T 15257A 15326G 15452A 16069T 16126C 16145A 16231C 16261T |  | NA |
|  | Mother - Bl (M199) |  |  | 73G 150T 152C 195C 215G 263G 295T 310.1T 315.1C 319C 489C 513A 750G 1438G 1850C 2706G 4216C 4375T 4769G 7028T 7476T 7789A 8860G 10398G 10499G 11251G 11377A 11719A 12612G 13708A 13722G 13830C 14133G 14766T 15257A 15326G 15452A 16069T 16126C 16145A 16231C 16261T |  | NA |
|  | Child - Bl (M199-C) |  |  | 73G 150T 152C 195C 215G 263G 295T 310.1T 315.1C 319C 489C 513A 750G 1438G 1850C 2706G 4216C 4375T 4769G 7028T 7476T 7789A 8860G 10398G 10499G 11251G 11377A 11719A 12612G 13708A 13722G 13830C 14133G 14766T 15257A 15326G 15452A 16069T 16126C 16145A 16231C 16261T |  | NA |
|  |  |  |  |  |  |  |
| 28 | Mother - Bu (739) |  | T596C (15.18%) | 263G **309.1C 309.2C 315.1C** 709A 750G 2259T 4769G 8266G 8860G 9548A 13762G 14066T 14872T 15217A 15326G | H13a2b | NA |
|  | Child - Bu (725) |  |  | 263G **309.1C 309.2C 315.1C** 709A 750G 2259T 4769G 8266G 8860G 9548A 13762G 14066T 14872T 15217A 15326G |  | NA |
|  | Mother - Bl (M200) |  | T596C (4.80%) | 263G 709A 750G 2259T 4769G 8266G 8860G 9548A 13762G 14066T 14872T 15217A 15326G |  | 309.1C 309.2C 315.1C below coverage threshold of 200 reads |
|  | Child - Bl (M200-C) |  | C11881T (2.07%) | 263G **309.1C 309.2C 315.1C** 709A 750G 2259T 4769G 8266G 8860G 9548A 13762G 14066T 14872T 15217A 15326G |  | NA |
|  |  |  |  |  |  |  |
| 29 | Mother - Bu (637) |  |  | 263G 309.1C 315.1C 750G 1438G 4769G 4793G 8860G 11025C 15326G 16213A 16519C | H7H | NA |
|  | Child - Bu (655) |  |  | 263G 309.1C 315.1C 750G 1438G 4769G 4793G 8860G 11025C 15326G 16213A 16519C |  | NA |
|  | Mother - Bl (M231) |  |  | 263G 309.1C 315.1C 750G 1438G 4769G 4793G 8860G 11025C 15326G 16213A 16519C |  | NA |
|  | Child - Bl (M231-C) |  |  | 263G 309.1C 315.1C 750G 1438G 4769G 4793G 8860G 11025C 15326G 16213A 16519C |  | NA |
|  |  |  |  |  |  |  |
| 30 | Mother - Bu (605) |  | A926G (3.49%) & T16189C (5.09%) | 10C 263G **309.1C 309.2C 315.1C** 750G 1438G 3501G 4769G 6776C 8470G 8860G 15326G 16129A 16519C | H3af | NA |
|  | Child - Bu (619) |  | T10970C (4.32%) | 10C 263G **309.1C 309.2C 315.1C** 750G 1438G 3501G 4769G 6776C 8470G 8860G 15326G 16129A 16519C |  | NA |
|  | Mother - Bl (M240) |  | A926G (3.66%) | 10C 263G 750G 1438G 3501G 4769G 6776C 8470G 8860G 15326G 16129A 16519C |  | 309.1C 309.2C 315.1C below coverage threshold of 200 reads |
|  | Child - Bl (M240-C) |  |  | 10C 263G **309.1C 309.2C 315.1C** 750G 1438G 3501G 4769G 6776C 8470G 8860G 15326G 16129A 16519C |  | NA |
|  |  |  |  |  |  |  |
| 31 | Mother - Bu (632) | G15047A (21.08%) | A14573G (29.02%) | 73G 146C 152C 263G 315.1C 498del 750G 1189C 1438G 1811G 2706G 3480G 4092A 4769G 7028T 8860G 9006G 9055A 9698C 10398G 10550G 11299C 11467G 11719A 12308G 12372A 14002G 14040A 14167T 14766T 14798C 15326G 16224C 16311C 16320T 16519C | K1c2 | NA |
|  | Child - Bu (696) | G15047A (26.67%) | A214G (8.45%), T11299T (2.64%) & A11467A (2.76%) | 73G 146C 152C 263G 315.1C 498del 750G 1189C 1438G 1811G 2706G 3480G 4092A 4769G 7028T 8860G 9006G 9055A 9698C 10398G 10550G 11299C 11467G 11719A 12308G 12372A 14002G 14040A 14167T 14766T 14798C 15326G 16224C 16311C 16320T 16519C |  | NA |
|  | Mother - Bl (M236) | G15047A (19.47%) | A14573G (22.47%) | 73G 146C 152C 263G 315.1C 498del 750G 1189C 1438G 1811G 2706G 3480G 4092A 4769G 7028T 8860G 9006G 9055A 9698C 10398G 10550G 11299C 11467G 11719A 12308G 12372A 14002G 14040A 14167T 14766T 14798C 15326G 16224C 16311C 16320T 16519C |  | NA |
|  | Child - Bl (M236-C) | G15047A (28.22%) | A214G (3.10%) | 73G 146C 152C 263G 315.1C 498del 750G 1189C 1438G 1811G 2706G 3480G 4092A 4769G 7028T 8860G 9006G 9055A 9698C 10398G 10550G 11299C 11467G 11719A 12308G 12372A 14002G 14040A 14167T 14766T 14798C 15326G 16224C 16311C 16320T 16519C |  | NA |
|  |  |  |  |  |  |  |
| 32 | Mother - Bu (531) | C5107T (9.74%) |  | 263G 315.1C 750G 1438G 3333T 4769G 7083G 8860G 15326G 16293G 16519C | H24a | NA |
|  | Child - Bu (572) | C5107T (13.07%) | A16240G (9.04%) | 263G 315.1C 750G 1438G 3333T 4769G 7083G 8860G 15326G 16293G 16519C |  | NA |
|  | Mother - Bl (M-188) | C5107T (8.19%) |  | 263G 315.1C 750G 1438G 3333T 4769G 7083G 8860G 15326G 16293G 16519C |  | NA |
|  | Child - Bl (M188-C) | C5107T (10.05%) | A16240G (5.61%) | 263G 315.1C 750G 1438G 3333T 4769G 7083G 8860G 15326G 16293G 16519C |  | NA |
|  |  |  |  |  |  |  |
| 33 | Mother - Bu (616) | T15262C (8.36%) |  | 263G 315.1C 477C 750G 1438G 3010A 4769G 8803G 8860G 15326G 16519C | H1c5 | NA |
|  | Child - Bu (643) | T15262C (15.81%) | T2559C (2.90%) & A9983G (2.56%) | 263G 315.1C 477C 750G 1438G 3010A 4769G 8803G 8860G 15326G 16519C |  | NA |
|  | Mother - Bl (M252) | T15262C (7.46%) |  | 263G 315.1C 477C 750G 1438G 3010A 4769G 8803G 8860G 15326G 16519C |  | NA |
|  | Child - Bl (M252-C) | T15262C (15.49%) | A9983G (2.02%) | 263G 315.1C 477C 750G 1438G 3010A 4769G 8803G 8860G 15326G 16519C |  | NA |
|  |  |  |  |  |  |  |
| 34 | Mother - Bu (826) |  |  | 73G 263G 315.1C 750G 1438G 3010A 4769G 6365C 8860G 15326G 16148T 16162G 16209C 16519C | H1a1 | NA |
|  | Child - Bu (816) |  |  | 73G 263G 315.1C 750G 1438G 3010A 4769G 6365C 8860G 15326G 16148T 16162G 16209C 16519C |  | NA |
|  | Mother - Bl (M486) |  |  | 73G 263G 315.1C 750G 1438G 3010A 4769G 6365C 8860G 15326G 16148T 16162G 16209C 16519C |  | NA |
|  | Child - Bl (M486-C) |  |  | 73G 263G 315.1C 750G 1438G 3010A 4769G 6365C 8860G 15326G 16148T 16162G 16209C 16519C |  | NA |
|  |  |  |  |  |  |  |
| 35 | Mother - Bu (1126) |  | T1391C (3.27%) & A2706A (2.69%) | 73G 263G 309.1C 315.1C 750G 1438G 2706G 3348G 4769G 7028T 7805A 8860G 10364A 11467G 11719A 12308G 12372A 14179G 14562T 14766T 15326G 15884A 16219G 16235G 16278T | U6a1b2 | NA |
|  | Child - Bu (1086) |  | T310C (3.38%) | 73G 263G 309.1C 315.1C 750G 1438G 2706G 3348G 4769G 7028T 7805A 8860G 10364A 11467G 11719A 12308G 12372A 14179G 14562T 14766T 15326G 15884A 16219G 16235G 16278T |  | NA |
|  | Mother - Bl (M513) |  |  | 73G 263G 309.1C 315.1C 750G 1438G 2706G 3348G 4769G 7028T 7805A 8860G 10364A 11467G 11719A 12308G 12372A 14179G 14562T 14766T 15326G 15884A 16219G 16235G 16278T |  | NA |
|  | Child - Bl (M513-C) |  |  | 73G 263G 309.1C 315.1C 750G 1438G 2706G 3348G 4769G 7028T 7805A 8860G 10364A 11467G 11719A 12308G 12372A 14179G 14562T 14766T 15326G 15884A 16219G 16235G 16278T |  | NA |
|  |  |  |  |  |  |  |
| 36 | Mother - Bu (1282) |  |  | 73G 150T 241G 263G 309.1C 315.1C 515G 524.1A 524.2C 524.3A 524.4C 750G 1095C 1391C 1438G 2706G 3394C 4769G 6683T 7028T 7805A 8860G 10410C 11122A 11151T 11224G 11719A 11809C 12245C 12358G 12406A 13167G 13359A 14239T 14766T 15326G 15884A 15940C 16311C 16390A 16519C | R1b1 | NA |
|  | Child - Bu (1159) |  |  | 73G 150T 241G 263G 309.1C 315.1C 515G 524.1A 524.2C 524.3A 524.4C 750G 1095C 1391C 1438G 2706G 3394C 4769G 6683T 7028T 7805A 8860G 10410C 11122A 11151T 11224G 11719A 11809C 12245C 12358G 12406A 13167G 13359A 14239T 14766T 15326G 15884A 15940C 16311C 16390A 16519C |  | NA |
|  | Mother - Bl (SC14) |  |  | 73G 150T 241G 263G 309.1C 315.1C 515G 524.1A 524.2C 524.3A 524.4C 750G 1095C 1391C 1438G 2706G 3394C 4769G 6683T 7028T 7805A 8860G 10410C 11122A 11151T 11224G 11719A 11809C 12245C 12358G 12406A 13167G 13359A 14239T 14766T 15326G 15884A 15940C 16311C 16390A 16519C |  | NA |
|  | Child - Bl (SC14-C) |  |  | 73G 150T 241G 263G 309.1C 315.1C 515G 524.1A 524.2C 524.3A 524.4C 750G 1095C 1391C 1438G 2706G 3394C 4769G 6683T 7028T 7805A 8860G 10410C 11122A 11151T 11224G 11719A 11809C 12245C 12358G 12406A 13167G 13359A 14239T 14766T 15326G 15884A 15940C 16311C 16390A 16519C |  | NA |
|  |  |  |  |  |  |  |
| 37 | Mother - Bu (1269) | A8289ins (CCCCCTCTA)(25.72%) |  | 263G 309.1C 315.1C 750G 1438G 2706G 4216C 4769G 7028T 8860G 13449T 15326G 16311C | HV10 | NA |
|  | Child - Bu (1169) | A8289ins (CCCCCTCTA)(25.52%) |  | 263G 309.1C 315.1C 750G 1438G 2706G 4216C 4769G 7028T 8860G 13449T 15326G 16311C |  | NA |
|  | Mother - Bl (SC8) | A8289ins (CCCCCTCTA)(25.97%) |  | 263G 309.1C 315.1C 750G 1438G 2706G 4216C 4769G 7028T 8860G 13449T 15326G 16311C |  | NA |
|  | Child - Bl (SC8-C) | A8289ins (CCCCCTCTA)(14.54%) |  | 263G 309.1C 315.1C 750G 1438G 2706G 4216C 4769G 7028T 8860G 13449T 15326G 16311C |  | NA |
|  |  |  |  |  |  |  |
| 38 | Mother - Bu (521) |  | A215G (6.45%) | 263G 477C 750G 1438G 3010A 4769G 5821A 8860G 9150G 15326G 16193T 16263C 16519C | H1c1 | NA |
|  | Child - Bu (548) |  | A16482G (6.7%) | 263G 477C 750G 1438G 3010A 4769G 5821A 8860G 9150G 15326G 16193T 16263C 16519C |  | NA |
|  | Mother - Bl (M190) |  | T5105C (2.41%) | 263G 477C 750G 1438G 3010A 4769G 5821A 8860G 9150G 15326G 16193T 16263C 16519C |  | NA |
|  | Child - Bl (M190-C) |  |  | 263G 477C 750G 1438G 3010A 4769G 5821A 8860G 9150G 15326G 16193T 16263C 16519C |  | NA |
|  |  |  |  |  |  |  |
| 39 | Mother - Bu (M250) |  |  | 73G 146C 152C 263G 315.1C 373G 498del 750G 1189C 1438G 1811G 2706G 3480G 4769G 7028T 8860G 9055A 9093G 9300A 9698C 10398G 10550G 11299C 11377A 11467G 11719A 12308G 12372A 14167T 14766T 14798C 15326G 16218T 16224C 16311C 16519C | K1c1 | NA |
|  | Child - Bu (M250C1) |  | A234G (3.96%) | 73G 146C 152C 263G 315.1C 373G 498del 750G 1189C 1438G 1811G 2706G 3480G 4769G 7028T 8860G 9055A 9093G 9300A 9698C 10398G 10550G 11299C 11377A 11467G 11719A 12308G 12372A 14167T 14766T 14798C 15326G 16218T 16224C 16311C 16519C |  | NA |
|  | Mother - Bl (M250) |  |  | 73G 146C 152C 263G 315.1C 373G 498del 750G 1189C 1438G 1811G 2706G 3480G 4769G 7028T 8860G 9055A 9093G 9300A 9698C 10398G 10550G 11299C 11377A 11467G 11719A 12308G 12372A 14167T 14766T 14798C 15326G 16218T 16224C 16311C 16519C |  | NA |
|  | Child - Bl (M250C1) |  |  | 73G 146C 152C 263G 315.1C 373G 498del 750G 1189C 1438G 1811G 2706G 3480G 4769G 7028T 8860G 9055A 9093G 9300A 9698C 10398G 10550G 11299C 11377A 11467G 11719A 12308G 12372A 14167T 14766T 14798C 15326G 16218T 16224C 16311C 16519C |  | NA |
