# Supplementary material for: Deep-Coverage MPS Analysis of Heteroplasmic Variants within the mtGenome Allows for Frequent Differentiation of Maternal Relatives
Source: Genes (Basel). 2018 Feb 26;9(3):124. doi: 10.3390/genes9030124 (PMC5867845; doi:10.3390/genes9030124)
Supplement: Supplementary file 1 [file genes-09-00124-s001.zip › Supplemental-final/Table S2.docx]

**Supplemental Table 2**: Sites of shared, differentiating, and random heteroplasmy for 29 of the 39 pairs; nine of the remaining 10 pairs exhibited no heteroplasmy, while the tenth pair had a shared, nine bp insertion. Read coverage and distribution, gene annotation and protein coding changes are provided. Sites of differentiating heteroplasmy in both buccal and blood cells are highlighted in **BOLD** text. A total of 16 of the 21 differentiating sites are in the coding region of the mtgenome.

|  | **Nucleotide Position** | **Sample Number** | **Major Allele** | **Coverage (#For:#Rev Reads)** | **Major Frequency (%)** | **Minor Allele** | **Coverage (#For:#Rev Reads)** | **Minor Frequency (%)** | **Gene Annotation** | **Synonymous (Y or N)** | **Shared Heteroplasmy (%)** | **Gene Annotation** | **Synonymous (Y or N)** |
| --- | --- | --- | --- | --- | --- | --- | --- | --- | --- | --- | --- | --- | --- |
| 1 | G185G | Mother - Bu (807) | A | 2972:5534 | 97.06 | G | 88:166 | 2.89 | CR |  | A16183G (7.32%) | CR |  |
|  |  | Child - Bu (803) | A |  |  |  |  |  |  |  | A16183G (6.89%) |  |  |
|  |  | Mother - Bl (M490) | A |  |  |  |  |  |  |  | A16183G (2.81%) |  |  |
|  |  | Child - Bl (M490-C) | A |  |  |  |  |  |  |  | A16183G (2.46%) |  |  |
|  | T16092T | Mother - Bu (807) | C | 10853:13965 | 97.54 | T | 260:353 | 2.4 | CR |  |  |  |  |
|  |  | Child - Bu (803) | C |  |  |  |  |  |  |  |  |  |  |
|  |  | Mother - Bl (M490) | C |  |  |  |  |  |  |  |  |  |  |
|  |  | Child - Bl (M490-C) | C |  |  |  |  |  |  |  |  |  |  |
|  |  |  |  |  |  |  |  |  |  |  |  |  |  |
| 2 | T13581C | Mother - Bu (618) | T |  |  |  |  |  |  |  | T16189C (7.74%) | CR |  |
|  |  | Child - Bu (606) | T | 5970:13041 | 94.53 | C | 194:889 | 5.38 | ND5 | Y (Ala) | T16189C (11.07%) |  |  |
|  |  | Mother - Bl (M249) | T |  |  |  |  |  |  |  | T16189C (2.81%) |  |  |
|  |  | Child - Bl (M249-C) | T |  |  |  |  |  |  |  | T16189C (9.92%) |  |  |
|  | T16093T | Mother - Bu (618) | C | 5913:10192 | 88.34 | T | 674:1426 | 11.52 | CR |  |  |  |  |
|  |  | Child - Bu (606) | C |  |  |  |  |  |  |  |  |  |  |
|  |  | Mother - Bl (M249) | C |  |  |  |  |  |  |  |  |  |  |
|  |  | Child - Bl (M249-C) | C |  |  |  |  |  |  |  |  |  |  |
|  |  |  |  |  |  |  |  |  |  |  |  |  |  |
| 3 | T2746C | Mother - Bu (693) | T | 2920:6014 | 79.67 | C | 655:1600 | 20.11 | 16S |  |  |  |  |
|  |  | Child - Bu (677) | T |  |  |  |  |  |  |  |  |  |  |
|  |  | Mother - Bl (M207) | T | 14187:14328 | 80.3 | C | 3440:3528 | 19.62 | 16S |  |  |  |  |
|  |  | Child - Bl (M207-C) | T |  |  |  |  |  |  |  |  |  |  |
|  |  |  |  |  |  |  |  |  |  |  |  |  |  |
| 4 | C16320T | Mother - Bu (406) | C | 4918:3843 | 72.33 | T | 1866:1474 | 27.57 | CR |  |  |  |  |
|  |  | Child - Bu (444) | C |  |  |  |  |  |  |  |  |  |  |
|  |  | Mother - Bl (M137) | C | 5412:4619 | 94.9 | T | 288:248 | 5.07 | CR |  |  |  |  |
|  |  | Child - Bl (M137-C) | C |  |  |  |  |  |  |  |  |  |  |
|  |  |  |  |  |  |  |  |  |  |  |  |  |  |
| 5 | G11825A | Mother - Bu (762) | G |  |  |  |  |  |  |  | T10873C (2.53%) | ND4 | Y (Pro) |
|  |  | Child - Bu (702) | G |  |  |  |  |  |  |  | T10873C (6.66%) |  |  |
|  |  | Mother - Bl (M210) | G | 5493:5624 | 97.78 | A | 121:124 | 2.15 | ND4 | N (Ala to Thr) |  |  |  |
|  |  | Child - Bl (M210-C) | G |  |  |  |  |  |  |  | T10873C (5.40%) |  |  |
|  |  |  |  |  |  |  |  |  |  |  |  |  |  |
| 6 | T9179C | Mother - Bu (1134) | T | 3063:5076 | 85.02 | C | 538:892 | 14.93 | ATP6 | N (Val to Ala) |  |  |  |
|  |  | Child - Bu (1099) | T |  |  |  |  |  |  |  |  |  |  |
|  |  | Mother - Bl (M502G) | T | 16583:20269 | 87.14 | C | 2468:2934 | 12.77 | ATP6 | N (Val to Ala) |  |  |  |
|  |  | Child - Bl (M501) | T |  |  |  |  |  |  |  |  |  |  |
|  | T16189C | Mother - Bu (1134) | T | 14423:17971 | 97.07 | C | 345:517 | 2.58 | CR |  |  |  |  |
|  |  | Child - Bu (1099) | T |  |  |  |  |  |  |  |  |  |  |
|  |  | Mother - Bl (M502G) | T |  |  |  |  |  |  |  |  |  |  |
|  |  | Child - Bl (M501) | T |  |  |  |  |  |  |  |  |  |  |
|  | C10980T | Mother - Bu (1134) | C |  |  |  |  |  |  |  |  |  |  |
|  |  | Child - Bu (1099) | C | 8429:4098 | 95.47 | T | 434:149 | 4.44 | ND4 | N (Pro to Leu) |  |  |  |
|  |  | Mother - Bl (M502G) | C |  |  |  |  |  |  |  |  |  |  |
|  |  | Child - Bl (M501) | C |  |  |  |  |  |  |  |  |  |  |
|  |  |  |  |  |  |  |  |  |  |  |  |  |  |
| 7 | C1948T | Mother - Bu (875) | C | 18116:12246 | 97.85 | T | 397:243 | 2.06 | 16S |  |  |  |  |
|  |  | Child - Bu (825) | C |  |  |  |  |  |  |  |  |  |  |
|  |  | Mother - Bl (M493) | C |  |  |  |  |  |  |  |  |  |  |
|  |  | Child - Bl (M493-C) | C |  |  |  |  |  |  |  |  |  |  |
|  |  |  |  |  |  |  |  |  |  |  |  |  |  |
| 8 | G14040A | Mother - Bu (659) | G | 5770:4227 | 92.01 | A | 474:381 | 7.86 | ND5 | Y (Gln) |  |  |  |
|  |  | Child - Bu (722) | G |  |  |  |  |  |  |  |  |  |  |
|  |  | Mother - Bl (M242) | G | 13200:12992 | 94.07 | A | 831:811 | 5.89 | ND5 | Y (Gln) |  |  |  |
|  |  | Child - Bl (M242-C) | G |  |  |  |  |  |  |  |  |  |  |
|  |  |  |  |  |  |  |  |  |  |  |  |  |  |
| 9 | T14461C | Mother - Bu (411) | T | 7078:7720 | 97.04 | C | 205:233 | 2.87 | ND6 | Y (Thr) |  |  |  |
|  |  | Child - Bu (401) | T |  |  |  |  |  |  |  |  |  |  |
|  |  | Mother - Bl (M132) | T | 8475:8875 | 97.54 | C | 193:237 | 2.41 | ND6 | Y (Thr) |  |  |  |
|  |  | Child - Bl (M132-C) | T |  |  |  |  |  |  |  |  |  |  |
|  |  |  |  |  |  |  |  |  |  |  |  |  |  |
| 10 | G11825A | Mother - Bu (711) | G | 1622:2662 | 93.41 | A | 116:184 | 6.54 | ND4 | N (Ala to Thr) |  |  |  |
|  |  | Child - Bu (737) | G |  |  |  |  |  |  |  |  |  |  |
|  |  | Mother - Bl (M203) | G | 4728:5871 | 97.1 | A | 133:167 | 2.74 | ND4 | N (Ala to Thr) |  |  |  |
|  |  | Child - Bl (M203-C) | G |  |  |  |  |  |  |  |  |  |  |
|  | T11616C | Mother - Bu (711) | T |  |  |  |  |  |  |  |  |  |  |
|  |  | Child - Bu (737) | T | 16082:13696 | 93.99 | C | 560:1320 | 5.93 | ND4 | N (Ile to Thr) |  |  |  |
|  |  | Mother - Bl (M203) | T |  |  |  |  |  |  |  |  |  |  |
|  |  | Child - Bl (M203-C) | T |  |  |  |  |  |  |  |  |  |  |
|  | T12375C | Mother - Bu (711) | T |  |  |  |  |  |  |  |  |  |  |
|  |  | Child - Bu (737) | T | 6368:6238 | 72.03 | C | 2770:2099 | 27.82 | ND5 | Y (Thr) |  |  |  |
|  |  | Mother - Bl (M203) | T |  |  |  |  |  |  |  |  |  |  |
|  |  | Child - Bl (M203-C) | T | 10455:10132 | 76 | C | 3481:3008 | 23.95 | ND5 | Y (Thr) |  |  |  |
|  | G9907A | Mother - Bu (711) | G |  |  |  |  |  |  |  |  |  |  |
|  |  | Child - Bu (737) | G |  |  |  |  |  |  |  |  |  |  |
|  |  | Mother - Bl (M203) | G |  |  |  |  |  |  |  |  |  |  |
|  |  | Child - Bl (M203-C) | G | 13008:20207 | 97.64 | A | 296:454 | 2.2 | CO3 | N (Gly to Asp) |  |  |  |
|  |  |  |  |  |  |  |  |  |  |  |  |  |  |
| 11 | T16093T | Mother - Bu (729) | C | 1917:3608 | 95.83 | T | 66:172 | 4.12 | CR |  | A1656A (2.11%) | tRNA^val^ |  |
|  |  | Child - Bu (684) | C |  |  |  |  |  |  |  | A1656A (2.52%) |  |  |
|  |  | Mother - Bl (M213) | C |  |  |  |  |  |  |  | A1656A (2.77%) |  |  |
|  |  | Child - Bl (M213-C) | C |  |  |  |  |  |  |  | A1656A (2.68%) |  |  |
|  | A13790G | Mother - Bu (729) | A |  |  |  |  |  |  |  |  |  |  |
|  |  | Child - Bu (684) | A | 5501:2427 | 88.46 | G | 650:356 | 11.22 | ND5 | N (Tyr to Cys) |  |  |  |
|  |  | Mother - Bl (M213) | A |  |  |  |  |  |  |  |  |  |  |
|  |  | Child - Bl (M213-C) | A | 5900:4359 | 88.44 | G | 705:583 | 11.1 | ND5 | N (Tyr to Cys) |  |  |  |
|  | A11362G | Mother - Bu (729) | A |  |  |  |  |  |  |  |  |  |  |
|  |  | Child - Bu (684) | A |  |  |  |  |  |  |  |  |  |  |
|  |  | Mother - Bl (M213) | A |  |  |  |  |  |  |  |  |  |  |
|  |  | Child - Bl (M213-C) | A | 38517:45495 | 97.84 | G | 911:905 | 2.11 | ND4 | Y (Met) |  |  |  |
|  |  |  |  |  |  |  |  |  |  |  |  |  |  |
| 12 | A16183C | Mother - Bu (1091) | A |  |  |  |  |  |  |  | A3243G (30.72%), A5539A (41.94%) & C16192C (19.23%) | tRNA^leu^, tRNA^trp^, CR |  |
|  |  | Child - Bu (1111) | A | 1999:2620 | 97.69 | C | 25:77 | 2.15 | CR |  | A3243A (33.10%), A5539G (24.54%) & C16192C (14.10%) |  |  |
|  |  | Mother - Bl (M512) | A |  |  |  |  |  |  |  | A3243G (13.13%), A5539A (23.13%) & C16192C (22.78%) |  |  |
|  |  | Child - Bl (M512-C) | A |  |  |  |  |  |  |  | A3243A (41.01%), A5539G (31.26%) & C16192C (17.30%) |  |  |
|  |  |  |  |  |  |  |  |  |  |  |  |  |  |
| 13 | A200A | Mother - Bu (1098) | G | 1350:3677 | 96.58 | A | 31:139 | 3.26 | CR |  | T16093C (11.53%) | CR |  |
|  |  | Child - Bu (1100) | G |  |  |  |  |  |  |  | T16093T (3.45%) |  |  |
|  |  | Mother - Bl (M520) | G | 821:1294 | 97.6 | A | 17:32 | 2.26 | CR |  | T16093C (9.12%) |  |  |
|  |  | Child - Bl (M520-C) | G |  |  |  |  |  |  |  |  |  |  |
|  |  |  |  |  |  |  |  |  |  |  |  |  |  |
| 14 | A215G | Mother - Bu (800) | A |  |  |  |  |  |  |  |  |  |  |
|  |  | Child - Bu (871) | A | 5744:9241 | 96.08 | G | 192:393 | 3.75 | CR |  |  |  |  |
|  |  | Mother - Bl (M480) | A |  |  |  |  |  |  |  |  |  |  |
|  |  | Child - Bl (M480-C) | A |  |  |  |  |  |  |  |  |  |  |
|  |  |  |  |  |  |  |  |  |  |  |  |  |  |
| 15 | A214G | Mother - Bu (1122) | A | 4842:9077 | 97.46 | G | 107:246 | 2.47 | CR |  |  |  |  |
|  |  | Child - Bu (1119) | A |  |  |  |  |  |  |  |  |  |  |
|  |  | Mother - Bl (M500) | A |  |  |  |  |  |  |  |  |  |  |
|  |  | Child - Bl (M500-C) | A |  |  |  |  |  |  |  |  |  |  |
|  | T310C | Mother - Bu (1122) | T | 15:4095 | 92.56 | C | 1:325 | 7.34 | CR |  |  |  |  |
|  |  | Child - Bu (1119) | T |  |  |  |  |  |  |  |  |  |  |
|  |  | Mother - Bl (M500) | T |  |  |  |  | (4.01) |  |  |  |  |  |
|  |  | Child - Bl (M500-C) | T |  |  |  |  |  |  |  |  |  |  |
|  | A4191T | Mother - Bu (1122) | A |  |  |  |  |  |  |  |  |  |  |
|  |  | Child - Bu (1119) | A | 4452:5729 | 95.5 | T | 202:243 | 4.17 | ND1 | Y (Pro) |  |  |  |
|  |  | Mother - Bl (M500) | A |  |  |  |  |  |  |  |  |  |  |
|  |  | Child - Bl (M500-C) | A | 12277:14284 | 94.96 | T | 612:695 | 4.67 | ND1 | Y (Pro) |  |  |  |
|  |  |  |  |  |  |  |  |  |  |  |  |  |  |
| 16 | T310C | Mother - Bu (1267) | T | 54:5184 | 91.15 | C | 3:450 | 7.88 | CR |  | T2352T (48.11%) | 16S |  |
|  |  | Child - Bu (1160) | T |  |  |  |  |  |  |  | T2352T (26.81%) |  |  |
|  |  | Mother - Bl (SC16) | T |  |  |  |  |  |  |  | T2352T (47.93%) |  |  |
|  |  | Child - Bl (SC16-C) | T |  |  |  |  |  |  |  | T2352T (26.84%) |  |  |
|  | G11149A | Mother - Bu (1267) | G | 55588:30794 | 97.76 | A | 1258:658 | 2.16 | ND4 | Y (Leu) |  |  |  |
|  |  | Child - Bu (1160) | G |  |  |  |  |  |  |  |  |  |  |
|  |  | Mother - Bl (SC16) | G |  |  |  |  |  |  |  |  |  |  |
|  |  | Child - Bl (SC16-C) | G |  |  |  |  |  |  |  |  |  |  |
|  | A16170G | Mother - Bu (1267) | A | 17593:21027 | 94.49 | G | 1060:1174 | 5.46 | CR |  |  |  |  |
|  |  | Child - Bu (1160) | A |  |  |  |  |  |  |  |  |  |  |
|  |  | Mother - Bl (SC16) | A | 8155:10691 | 96.19 | G | 332:413 | 3.8 | CR |  |  |  |  |
|  |  | Child - Bl (SC16-C) | A |  |  |  |  |  |  |  |  |  |  |
|  |  |  |  |  |  |  |  |  |  |  |  |  |  |
| 17 | T16172C | Mother - Bu (508) | T |  |  |  |  |  |  |  |  |  |  |
|  |  | Child - Bu (544) | T |  |  |  |  |  |  |  |  |  |  |
|  |  | Mother - Bl (M196) | T | 14113:17748 | 95.64 | C | 644:713 | 4.07 | CR |  |  |  |  |
|  |  | Child - Bl (M196-C) | T |  |  |  |  |  |  |  |  |  |  |
|  |  |  |  |  |  |  |  |  |  |  |  |  |  |
| 18 | T310C | Mother - Bu (589) | T | 15:2956 | 93.72 | C | 1:169 | 5.36 | CR |  |  |  |  |
|  |  | Child - Bu (491) | T |  |  |  |  |  |  |  |  |  |  |
|  |  | Mother - Bl (M186) | T |  |  |  |  |  |  |  |  |  |  |
|  |  | Child - Bl (M186-C) | T |  |  |  |  |  |  |  |  |  |  |
|  |  |  |  |  |  |  |  |  |  |  |  |  |  |
| 19 | T195C | Mother - Bu (839) | T | 3215:6908 | 93.15 | C | 221:510 | 6.72 | CR |  | C11635T (8.34%) | ND4 | Y (Ser) |
|  |  | Child - Bu (1189) | T |  |  |  |  |  |  |  | C11635T (17.93%) |  |  |
|  |  | Mother - Bl (M494) | T |  |  |  |  |  |  |  | C11635T (7.23%) |  |  |
|  |  | Child - Bl (M494-C) | T |  |  |  |  |  |  |  | C11635T (19.88%) |  |  |
|  | T310C | Mother - Bu (839) | T | 33:3251 | 90.17 | C | 4:308 | 8.56 | CR |  |  |  |  |
|  |  | Child - Bu (1189) | T |  |  |  |  |  |  |  |  |  |  |
|  |  | Mother - Bl (M494) | T |  |  |  |  |  |  |  |  |  |  |
|  |  | Child - Bl (M494-C) | T |  |  |  |  |  |  |  |  |  |  |
|  | G9196A | Mother - Bu (839) | G | 6061:10535 | 97.36 | A | 172:265 | 2.56 | ATP6 | N (Asp to Asn) |  |  |  |
|  |  | Child - Bu (1189) | G |  |  |  |  |  |  |  |  |  |  |
|  |  | Mother - Bl (M494) | G | 14294:16248 | 97.83 | A | 306:362 | 2.13 | ATP6 | N (Asp to Asn) |  |  |  |
|  |  | Child - Bl (M494-C) | G |  |  |  |  |  |  |  |  |  |  |
|  | T3183C | Mother - Bu (839) | T |  |  |  |  |  |  |  |  |  |  |
|  |  | Child - Bu (1189) | T | 12210:26317 | 96.49 | C | 412:937 | 3.37 | 16S |  |  |  |  |
|  |  | Mother - Bl (M494) | T |  |  |  |  |  |  |  |  |  |  |
|  |  | Child - Bl (M494-C) | T | 30655:39617 | 96.78 | C | 970:1333 | 3.17 | 16S |  |  |  |  |
|  | A15948G | Mother - Bu (839) | A |  |  |  |  |  |  |  |  |  |  |
|  |  | Child - Bu (1189) | A | 21533:19721 | 95.35 | G | 1039:902 | 4.48 | tRNA^thr^ |  |  |  |  |
|  |  | Mother - Bl (M494) | A |  |  |  |  |  |  |  |  |  |  |
|  |  | Child - Bl (M494-C) | A | 30887:30673 | 96.64 | G | 1074:1041 | 3.32 | tRNA^thr^ |  |  |  |  |
|  |  |  |  |  |  |  |  |  |  |  |  |  |  |
| 20 | C11288T | Mother - Bu (740) | C |  |  |  |  |  |  |  |  |  |  |
|  |  | Child - Bu (718) | C | 68908:55901 | 95.69 | T | 3140:2418 | 4.26 | ND4 | Y (Leu) |  |  |  |
|  |  | Mother - Bl (M211) | C |  |  |  |  |  |  |  |  |  |  |
|  |  | Child - Bl (M211-C) | C | 46511:43582 | 96.58 | T | 1651:1523 | 3.4 | ND4 | Y (Leu) |  |  |  |
|  |  |  |  |  |  |  |  |  |  |  |  |  |  |
| 21 | T596C | Mother - Bu (739) | T | 3088:856 | 84.7 | C | 552:155 | 15.18 | tRNA^phe^ |  |  |  |  |
|  |  | Child - Bu (725) | T |  |  |  |  |  |  |  |  |  |  |
|  |  | Mother - Bl (M200) | T | 1745:1125 | 95.15 | C | 93:52 | 4.8 | tRNA^phe^ |  |  |  |  |
|  |  | Child - Bl (M200-C) | T |  |  |  |  |  |  |  |  |  |  |
|  | C11881T | Mother - Bu (739) | C |  |  |  |  |  |  |  |  |  |  |
|  |  | Child - Bu (725) | C |  |  |  |  |  |  |  |  |  |  |
|  |  | Mother - Bl (M200) | C |  |  |  |  |  |  |  |  |  |  |
|  |  | Child - Bl (M200-C) | C | 14891:17661 | 97.9 | T | 335:354 | 2.07 | ND4 | Y (Asn) |  |  |  |
|  |  |  |  |  |  |  |  |  |  |  |  |  |  |
| 22 | A926G | Mother - Bu (605) | A | 7528:4119 | 96.48 | G | 275:147 | 3.49 | 12S |  |  |  |  |
|  |  | Child - Bu (619) | A |  |  |  |  |  |  |  |  |  |  |
|  |  | Mother - Bl (M240) | A | 3882:3469 | 96.29 | G | 149:131 | 3.66 | 12S |  |  |  |  |
|  |  | Child - Bl (M240-C) | A |  |  |  |  |  |  |  |  |  |  |
|  | T16189C | Mother - Bu (605) | T | 4235:5959 | 94.62 | C | 209:340 | 5.09 | CR |  |  |  |  |
|  |  | Child - Bu (619) | T |  |  |  |  |  |  |  |  |  |  |
|  |  | Mother - Bl (M240) | T |  |  |  |  |  |  |  |  |  |  |
|  |  | Child - Bl (M240-C) | T |  |  |  |  |  |  |  |  |  |  |
|  | T10970C | Mother - Bu (605) | T |  |  |  |  |  |  |  |  |  |  |
|  |  | Child - Bu (619) | T | 60433:30409 | 95.58 | C | 3467:639 | 4.32 | ND4 | N (Trp to Arg) |  |  |  |
|  |  | Mother - Bl (M240) | T |  |  |  |  |  |  |  |  |  |  |
|  |  | Child - Bl (M240-C) | T |  |  |  |  |  |  |  |  |  |  |
|  |  |  |  |  |  |  |  |  |  |  |  |  |  |
| 23 | A14573G | Mother - Bu (632) | A | 3406:2340 | 70.79 | G | 1390:966 | 29.02 | ND6 | N (Val to Ala) | G15047A (21.08%) | CYB | N (Gly to Ser) |
|  |  | Child - Bu (696) | A |  |  |  |  |  |  |  | G15047A (26.67%) |  |  |
|  |  | Mother - Bl (M236) | A | 7546:6525 | 77.51 | G | 2240:1839 | 22.47 | ND6 | N (Val to Ala) | G15047A (19.47%) |  |  |
|  |  | Child - Bl (M236-C) | A |  |  |  |  |  |  |  | G15047A (28.22%) |  |  |
|  | A214G | Mother - Bu (632) | A |  |  |  |  |  |  |  |  |  |  |
|  |  | Child - Bu (696) | A | 1739:2434 | 91.43 | G | 156:230 | 8.45 | CR |  |  |  |  |
|  |  | Mother - Bl (M236) | A |  |  |  |  |  |  |  |  |  |  |
|  |  | Child - Bl (M236-C) | A | 3426:4146 | 96.87 | G | 119:124 | 3.1 | CR |  |  |  |  |
|  | T11299T | Mother - Bu (632) | C |  |  |  |  |  |  |  |  |  |  |
|  |  | Child - Bu (696) | C | 18403:16756 | 97.24 | T | 503:452 | 2.64 | ND4 | Y (Thr) |  |  |  |
|  |  | Mother - Bl (M236) | C |  |  |  |  |  |  |  |  |  |  |
|  |  | Child - Bl (M236-C) | C |  |  |  |  |  |  |  |  |  |  |
|  | A11467A | Mother - Bu (632) | G |  |  |  |  |  |  |  |  |  |  |
|  |  | Child - Bu (696) | G | 12802:17089 | 97.09 | A | 408:443 | 2.76 | ND4 | Y (Leu) |  |  |  |
|  |  | Mother - Bl (M236) | G |  |  |  |  |  |  |  |  |  |  |
|  |  | Child - Bl (M236-C) | G |  |  |  |  |  |  |  |  |  |  |
|  |  |  |  |  |  |  |  |  |  |  |  |  |  |
| 24 | A16240G | Mother - Bu (531) | A |  |  |  |  |  |  |  | C5107T (9.74%) | ND2 | N (Thr to Ile) |
|  |  | Child - Bu (572) | A | 24590:25415 | 90.81 | G | 2527:2455 | 9.04 | CR |  | C5107T (13.07%) |  |  |
|  |  | Mother - Bl (M-188) | A |  |  |  |  |  |  |  | C5107T (8.19%) |  |  |
|  |  | Child - Bl (M188-C) | A | 13732:14444 | 94.28 | G | 823:855 | 5.61 | CR |  | C5107T (10.05%) |  |  |
|  |  |  |  |  |  |  |  |  |  |  |  |  |  |
| 25 | T2559C | Mother - Bu (616) | T |  |  |  |  |  |  |  | T15262C (8.36%) | CYB | Y (Ser) |
|  |  | Child - Bu (643) | T | 49062:50022 | 97.03 | C | 1461:1507 | 2.9 | 16S |  | T15262C (15.81%) |  |  |
|  |  | Mother - Bl (M252) | T |  |  |  |  |  |  |  | T15262C (7.46%) |  |  |
|  |  | Child - Bl (M252-C) | T |  |  |  |  |  |  |  | T15262C (15.49%) |  |  |
|  | A9983G | Mother - Bu (616) | A |  |  |  |  |  |  |  |  |  |  |
|  |  | Child - Bu (643) | A | 11623:20991 | 97.33 | G | 314:547 | 2.56 | CO3 | Y (Trp) |  |  |  |
|  |  | Mother - Bl (M252) | A |  |  |  |  |  |  |  |  |  |  |
|  |  | Child - Bl (M252-C) | A | 17123:22272 | 97.91 | G | 346:468 | 2.02 | CO3 | Y (Trp) |  |  |  |
|  |  |  |  |  |  |  |  |  |  |  |  |  |  |
| 26 | T1391C | Mother - Bu (1126) | T | 6617:8818 | 96.61 | C | 220:303 | 3.27 | 12S |  |  |  |  |
|  |  | Child - Bu (1086) | T |  |  |  |  |  |  |  |  |  |  |
|  |  | Mother - Bl (M513) | T |  |  |  |  |  |  |  |  |  |  |
|  |  | Child - Bl (M513-C) | T |  |  |  |  |  |  |  |  |  |  |
|  | A2706A | Mother - Bu (1126) | G | 2720:6616 | 96.16 | A | 126:136 | 2.69 | 16S |  |  |  |  |
|  |  | Child - Bu (1086) | G |  |  |  |  |  |  |  |  |  |  |
|  |  | Mother - Bl (M513) | G |  |  |  |  |  |  |  |  |  |  |
|  |  | Child - Bl (M513-C) | G |  |  |  |  |  |  |  |  |  |  |
|  | T310C | Mother - Bu (1126) | T |  |  |  |  |  |  |  |  |  |  |
|  |  | Child - Bu (1086) | T | 11:5129 | 96.59 | C | 1:179 | 3.38 | CR |  |  |  |  |
|  |  | Mother - Bl (M513) | T |  |  |  |  |  |  |  |  |  |  |
|  |  | Child - Bl (M513-C) | T |  |  |  |  |  |  |  |  |  |  |
|  |  |  |  |  |  |  |  |  |  |  |  |  |  |
| 27 | A215G | Mother - Bu (521) | A | 2086:5491 | 93.53 | G | 125:398 | 6.45 | CR |  |  |  |  |
|  |  | Child - Bu (548) | A |  |  |  |  |  |  |  |  |  |  |
|  |  | Mother - Bl (M190) | A |  |  |  |  |  |  |  |  |  |  |
|  |  | Child - Bl (M190-C) | A |  |  |  |  |  |  |  |  |  |  |
|  | A16482G | Mother - Bu (521) | A |  |  |  |  |  |  |  |  |  |  |
|  |  | Child - Bu (548) | A | 8065:7024 | 93.27 | G | 567:518 | 6.70 | CR |  |  |  |  |
|  |  | Mother - Bl (M190) | A |  |  |  |  |  |  |  |  |  |  |
|  |  | Child - Bl (M190-C) | A |  |  |  |  |  |  |  |  |  |  |
|  | T5105C | Mother - Bu (521) | T |  |  |  |  |  |  |  |  |  |  |
|  |  | Child - Bu (548) | T |  |  |  |  |  |  |  |  |  |  |
|  |  | Mother - Bl (M190) | T | 3880:3122 | 97.58 | C | 97:76 | 2.41 | ND2 | Y (Thr) |  |  |  |
|  |  | Child - Bl (M190-C) | T |  |  |  |  |  |  |  |  |  |  |
|  |  |  |  |  |  |  |  |  |  |  |  |  |  |
| 28 | A234G | Mother - Bu (M250) | A |  |  |  |  |  |  |  |  |  |  |
|  |  | Child - Bu (M250C1) | A | 1105:1265 | 95.99 | G | 43:55 | 3.96 | CR |  |  |  |  |
|  |  | Mother - Bl (M250) | A |  |  |  |  |  |  |  |  |  |  |
|  |  | Child - Bl (M250C1) | A |  |  |  |  |  |  |  |  |  |  |
|  |  |  |  |  |  |  |  |  |  |  |  |  |  |
|  |  |  |  |  |  |  |  |  |  |  |  |  |  |
|  | Shared Heteroplasmy Only: | | | |  |  |  |  |  |  |  |  |  |
|  |  |  |  |  |  |  |  |  |  |  |  |  |  |
| 29 |  | Mother - Bu (704) |  |  |  |  |  |  |  |  | T6152C (7.23%) | CO1 | Y (Val) |
|  |  | Child - Bu (630) |  |  |  |  |  |  |  |  | T6152C (16.37%) |  |  |
|  |  | Mother - Bl (M234) |  |  |  |  |  |  |  |  | T6152C (5.04%) |  |  |
|  |  | Child - Bl (M234-C) |  |  |  |  |  |  |  |  | T6152C (16.48%) |  |  |
|  |  |  |  |  |  |  |  |  |  |  |  |  |  |
