# Supplementary material for: Deep-Coverage MPS Analysis of Heteroplasmic Variants within the mtGenome Allows for Frequent Differentiation of Maternal Relatives
Source: Genes (Basel). 2018 Feb 26;9(3):124. doi: 10.3390/genes9030124 (PMC5867845; doi:10.3390/genes9030124)
Supplement: Supplementary file 1 [file genes-09-00124-s001.zip › Supplemental-final/Table S3.docx]

**Supplemental Table 3**: Comparison of samples with differentiating heteroplasmy, shared heteroplasmy and random sites of heteroplasmy that are neither shared nor differentiating. No duplicate sites were observed within the datasets for differentiating and shared heteroplasmy, with one site (16093) observed in both the differentiating and shared datasets. A total of 28 of the 96 differentiating and shared sites (29%, buccal and blood combined) have frequencies above 10%. Each range of heteroplasmy is reported from blood (Bl) to buccal (Bu), not lowest to highest value.

| Sample No. | Differentiating Hetero | Percentage Range of Hetero | Sample No. | Shared Hetero | Percentage Range of Hetero (Mother & Child) | Sample No. | Non-Shared or Non-Differentiating Hetero | Percentage of Hetero | Tissue Type |
| --- | --- | --- | --- | --- | --- | --- | --- | --- | --- |
| 1098 M520 | A200A | 2.26-3.26% | 729 **684** M213 **M213-C** | A1656A | M 2.77-2.11% | **807** | G185G | 2.89% | Bu |
| **696 M236-C** | A214G | 3.10-8.45% |  |  | C 2.68-2.52% | 839 | T195C | 6.72% | Bu |
| 739 M200 | T596C | 4.80-15.18% | **1267** 1160 **SC16** SC16-C | T2352T | M 47.93-48.11% | 1122 | A214G | 2.47% | Bu |
| 605 M240 | A926G | 3.66-3.49% |  |  | C 26.84-26.81 | 871 | A215G | 3.75% | Bu |
| 693 M207 | T2746C | 19.62-20.11% | **1091 1111 M512 M512-C** | A3243G | M 13.13-30.72% | 521 | A215G | 6.45% | Bu |
| **1189 M494-C** | T3183C | 3.17-3.37% |  |  | C 41.01-33.10% | M250C1 | A234G | 3.96% | Bu |
| 1119 M500-C | A4191T | 4.67-4.17% | 531 **572** M188 **M188-C** | C5107T | M 8.19-9.74% | **1126** | T1391C | 3.27% | Bu |
| 1134 M502G | T9179C | 12.77-14.93% |  |  | C 10.05-13.07% | 875 | C1948T | 2.06% | Bu |
| **839 M494** | G9196A | 2.13-2.56% | **1091 1111 M512 M512-C** | A5539A | M 23.13-41.94% | 643 | T2559C | 2.90% | Bu |
| 643 M252-C | A9983G | 2.02-2.56% |  |  | C 31.26-24.54% | **1126** | A2706A | 2.69% | Bu |
| 718 M211-C | C11288T | 3.40-4.26% | 704 630 M234 M234-C | T6152C | M 5.04-7.23% | M190 | T5105C | 2.41% | Bl |
| **711 M203** | G11825A | 2.74-6.54% |  |  | C 16.48-16.37% | M203-C | G9907A | 2.20% | Bl |
| **737 M203-C** | T12375C | 23.95-27.82% | 762 702 M210-C | T10873C | M 2.53% (Bu) | 619 | T10970C | 4.32% | Bu |
| 684 M213-C | A13790G | 11.10-11.22% |  |  | C 5.40-6.66% | 1099 | C10980T | 4.44% | Bu |
| 659 M242 | G14040A | 5.89-7.86% | **839** **1189** **M494** **M494-C** | C11635T | M 7.23-8.34% | 1267 | G11149A | 2.16% | Bu |
| 411 M132 | T14461C | 2.41-2.87% |  |  | C 19.88-17.93% | **696** | T11299T | 2.64% | Bu |
| **632 M236** | A14573G | 22.47-29.02% | **632** **696** **M236** **M236-C** | G15047A | M 19.47-21.08% | M213-C | A11362G | 2.11% | Bl |
| **1189 M494-C** | A15948G | 3.32-4.48% |  |  | C 28.22-26.67% | **696** | A11467A | 2.76% | Bu |
| 1267 SC16 | A16170G | 3.80-5.46% | 616 **643** M252 **M252-C** | T15262C | M 7.46-8.36% | 737 | T11616C | 5.93% | Bu |
| 572 M188-C | A16240G | 5.61-9.04% |  |  | C 15.49-15.81% | M210 | G11825A | 2.15% | Bl |
| 406 M137 | C16320T | 5.07-27.57% | **1098** 1100 **M520** | T16093C | M 9.12-11.53% | M200-C | C11881T | 2.07% | Bl |
|  |  |  |  |  | C 3.45% (Bu) | 606 | T13581C | 5.38% | Bu |
| 21 differentiating heteroplasmy sites (no duplicates)  9/21 child and 12/21 mother with differentiating heteroplasmy  19/21 have Bu>Bl and 2/21 Bl>Bu in relation to rates of heteroplasmy at the site  Families with multiple sites of differentiating heteroplasmy are in **colored** font  For 14/21 both tissues are <10% (67%), for 5/21 both tissues are >10% (24%), with 2/21 others (9%) | | |  |  |  | **807** | T16092T | 2.40% | Bu |
|  |  |  | 807 803 M490 M490-C | A16183G | M 2.81-7.32% | 618 | T16093T | 11.52% | Bu |
|  |  |  |  |  | C 2.46-6.89% | 729 | T16093T | 4.12% | Bu |
|  |  |  |  |  |  | M196 | T16172C | 4.07% | Bl |
|  |  |  | 618 606 M249 M249-C | T16189C | M 2.81-7.74% | 1111 | A16183C | 2.15% | Bu |
|  |  |  |  |  | C 9.92-11.07% | 1134 | T16189C | 2.58% | Bu |
|  |  |  |  |  |  | 605 | T16189C | 5.09% | Bu |
|  |  |  | **1091 1111 M512 M512-C** | C16192C | M 22.78-19.23% | 548 | A16482G | 6.70% | Bu |
|  |  |  |  |  | C 17.30-14.10% | 16/30 sites <3%, 1/30 sites >10%, 13/30 sites between 3-10%  6/30 sites observed in blood (Bl) samples, 24/30 sites observed in buccal (Bu) samples  Multiple sites of random heteroplasmy in a single individual are highlighted in **BOLD** font | | | |
|  |  |  | 14 shared sites (no duplicates)  1 duplicate between the datasets (16093)  For 3/14 all tissues are <10% (21%), for 5/14 all tissues are >10% (36%), with 6/14 others (43%)  Samples with differentiating heteroplasmy, in addition to shared heteroplasmy, are in **BOLD** font  Families with multiple sites of shared heteroplasmy are in **blue** font | | |  |  |  |  |
|  |  |  |  |  |  |  |  |  |  |
|  |  |  |  |  |  |  | | | |
